# KMT2A regulates the autophagy-GATA4 axis through METTL3-mediated m^6^A modification of ATG4a to promote NPCs senescence and IVDD progression

**DOI:** 10.1038/s41413-024-00373-1

**Published:** 2024-11-21

**Authors:** Ouqiang Wu, Yuxin Jin, Zhiguang Zhang, Hao Zhou, Wenbin Xu, Linjie Chen, Morgan Jones, Kenny Yat Hong Kwan, Jianyuan Gao, Kai Zhang, Xiaofei Cheng, Qizhu Chen, Xinzhou Wang, Yan Michael Li, Zhenyu Guo, Jing Sun, Zhihua Chen, Bin Wang, Xiangyang Wang, Shuying Shen, Aimin Wu

**Affiliations:** 1 Department of Orthopaedics, Key Laboratory of Structural Malformations in Children of Zhejiang Province, Key Laboratory of Orthopaedics of Zhejiang Province, The Second Affiliated Hospital and Yuying Children’s Hospital of Wenzhou Medical University, Wenzhou, Zhejiang China; 2 Department of Emergency Medicine Center, Jinhua Municipal Central Hospital, Zhejiang, China; 3 Department of Orthopaedics, The First Affiliated Hospital of Ningbo University, Ningbo, Zhejiang China; 4 Department of Orthopaedics, Key Laboratory of Musculoskeletal System Degeneration and Regeneration Translational Research of Zhejiang Province, Sir Run Shaw Hospital, Zhejiang University School of Medicine, Hangzhou, China; 5 Spine Unit, The Royal Orthopaedic Hospital, Bristol Road South, Northfield, Birmingham, UK; 6 Department of Orthopaedics and Traumatology, Li Ka Shing Faculty of Medicine, The University of Hong Kong, Pokfulam, Hong Kong; 7 Shanghai Key Laboratory of Orthopedic Implants, Department of Orthopedics, Ninth People’s Hospital, Shanghai Jiao Tong University School of Medicine, Shanghai, China; 8 Department of Neurosurgery, University of Rochester Medical Center, Rochester, NY USA; 9 Department of Orthopaedic Surgery, The First Affiliated Hospital, Zhejiang University School of Medicine, Hangzhou, China

**Keywords:** Bone, Bone quality and biomechanics

## Abstract

Intervertebral disc degeneration (IVDD), a disease associated with ageing, is characterised by a notable increase in senescent nucleus pulposus cells (NPCs) as IVDD progresses. However, the specific mechanisms that regulate the senescence of NPCs remain unknown. In this study, we observed impaired autophagy in IVDD-NPCs, which contributed to the upregulation of NPCs senescence and the senescence-associated secretory phenotype (SASP). The dysregulated SASP disrupted NPCs viability and initiated extracellular matrix degradation. Conversely, the restoration of autophagy reversed the senescence phenotype by inhibiting GATA binding protein 4 (GATA4). Moreover, we made the novel observation that a cross-talk between histone H3 lysine 4 trimethylation (H3K4me3) modification and N6-methyladenosine(m^6^A)-methylated modification regulates autophagy in IVDD-NPCs. Mechanistically, lysine methyltransferase 2A (KMT2A) promoted the expression of methyltransferase-like 3 (METTL3) through H3K4me3 modification, whereas METTL3-mediated m^6^A modification reduced the expression of autophagy-associated 4a (ATG4a) by attenuating its RNA stability, leading to autophagy damage in NPCs. Silencing KMT2A and METTL3 enhanced autophagic flux and suppressed SASP expression in IVDD-NPCs. Therefore, targeting the H3K4me3-regulated METTL3/ATG4a/GATA4 axis may represent a promising new therapeutic strategy for IVDD.

## Introduction

The prevalence and impact of age-dependent diseases increase with human longevity, and according to the Global Burden of Disease Study, low back pain is a significant cause of disability and limitation of daily living in adults worldwide.^1^ According to projections, more than 800 million people worldwide will suffer from low back pain by 2050.^2^ Although the aetiology of low back pain is multifactorial, Intervertebral Disc Degeneration (IVDD) is widely recognised as the most common degenerative disease causing low back pain.^3^ Whereas ageing exacerbates disc degeneration and disease progression while affecting the body’s metabolism,^4^ improving our understanding of how ageing contributes to the progression of IVDD will provide new strategies for slowing down or halting the progression of the disease, which may have significant public health implications.

IVDD is characterised by the loss of resident cells and extracellular matrix (ECM).^5^ Nucleus pulposus cells (NPCs) are the predominant cell type in the disc centre and play a crucial role in the production of ECM and maintenance of the disc microenvironment.^6^ Therefore, degenerative changes in the NP, especially in NPCs, are the main pathogenesis of IVDD.^7^ IVDD as an age-related chronic disease, is closely related to its pathogenesis with the prolonged presence of senescent cells. NPCs have various characteristics of senescent cells during ageing and IVDD progression.^8^ The main features of senescent cells are cell cycle arrest, anti-apoptosis, and catabolic factor production, and exhibit a strong senescence-associated secretory phenotype (SASP).^9^ SASP not only affects the cellular microenvironment but also accelerates the senescence process of surrounding cells,^10^ which overlaps with the mediators that promote the development of IVDD. However, the molecular mechanisms underlying the senescence of NPCs in IVDD remain unclear and further studies are needed to reveal their complexity.

As a key metabolic process that maintains cellular homoeostasis, autophagy regulates the removal of cellular components, including damaged proteins and dysfunctional organelles, by delivering them to the lysosome.^11,12^ There is now growing evidence that increased SASP expression and accelerated senescence processes are closely associated with autophagy inhibition.^13^ Autophagy in the NPCs is progressively weakened during the ageing process, thereby inducing the onset of senescence and exacerbating disc degeneration.^14–17^ However, the specific mechanism by which autophagy is impaired in IVDD still needs to be further elucidated.

N6-methyladenosine (m^6^A), a widespread post-transcriptional RNA modification in eukaryotic cells, is implicated in various biological and pathological processes.^18^ m^6^A modifications are critical in cellular functions, particularly in gene expression regulation, DNA damage repair, autophagy, and cellular senescence.^13,19,20^ These modifications are dynamic and reversible, with methylases adding and demethylases removing them.^21,22^ Specific ‘reader’ proteins recognise modified transcripts, influencing mRNA functionality, including translation and stability.^23^ Recent studies have shown that Wilms tumour 1-associating protein (WTAP), an essential component of the m^6^A methyltransferase complex, is significantly upregulated in degenerating NPs of human intervertebral discs; furthermore, WTAP knockdown proves beneficial in decelerating NPCs ageing.^24^ However, the biological significance of m^6^A modification and its regulatory mechanisms during IVDD have yet to be fully elucidated.

Epigenetic alterations associated with ageing include the regulation of non-coding RNAs, DNA methylation, histone modification, and chromatin remodelling.^25^ Among these, the regulation of histone modification and chromatin accessibility stands out in the ageing context.^26^ These modifications significantly influence gene expression by facilitating or inhibiting the opening of chromatin and subsequent transcription.^27^ Recent studies have revealed that interactions between histone modifications and other epigenetic processes (m^6^A, non-coding RNAs, and DNA methylation) are crucial for gene regulation, indicating a more complex layer of epigenetic control.^28–30^ However, the specific epigenetic contributions to IVDD, a condition closely linked to ageing, are not fully comprehended. Unravelling these mechanisms in NPCs is vital for a deeper insight into IVDD pathogenesis.

This study concentrates on the impact of m^6^A modifications in the ageing of NPCs and the progression of IVDD. Our findings indicate an upregulation of the m^6^A methyltransferase METTL3, driven by KMT2A-mediated H3K4me3 promoter modification. Subsequent research demonstrated that METTL3 modulates the stability of ATG4a mRNA, an autophagy-related gene, via an m^6^A-YTHDF2-dependent pathway, leading to compromised autophagic function in NPCs. The resulting autophagy impairment further propels NPCs senescence and IVDD, with the senescence-associated regulator GATA4 playing a pivotal role. These interlinked pathways suggest that the H3K4me3-METTL3/ATG4a/GATA4 axis could act as a promising IVDD therapeutic target.

## Results

### Senescence and autophagy impairment of NPCs are closely related to the progression of IVDD

**Fig. 1:**
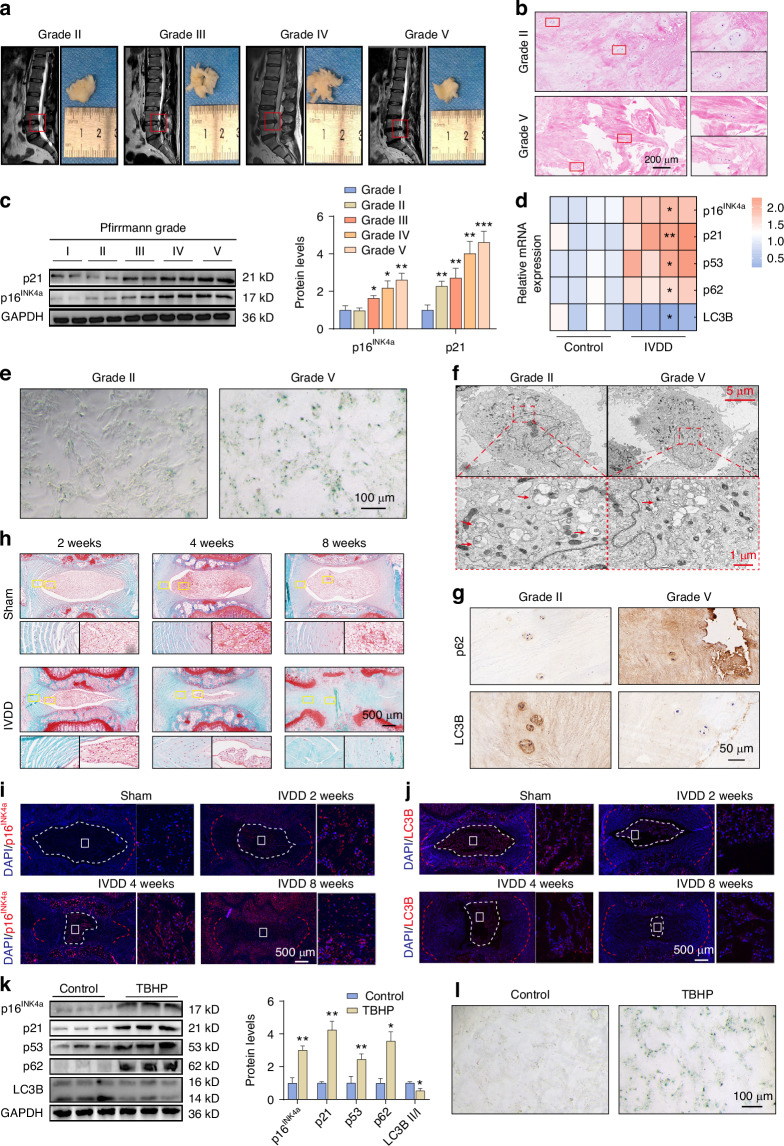
Senescence and autophagy impairment of NPCs are closely related to the progression of IVDD. **a** Representative Magnetic Resonance Imaging (MRI) was collected from patients exhibiting varying degrees of degeneration, classified according to the Pfirrmann MRI grading system. **b** HE staining of Grade II and Grade V NP tissues. **c** Western blot analyses were performed to determine the protein expression levels of senescence markers (p16^INK4a^, p21) in NP tissues from individuals with different degenerative grades (I, II, III, IV, V) (*n* = 3). *P < 0.05, **P < 0.01, ***P < 0.001. **d** qPCR assays were conducted on NP tissues from both normal and degenerated patients to measure mRNA levels of p16^INK4a^, p21, p53, p62, and LC3B (*n* = 3). *P < 0.05, **P < 0.01. **e** Number of positive cells in human NPCs of Grade II and Grade V origin detected by SA-β-gal. **f** Representative transmission electron microscopy images were acquired of human normal and degenerated NPCs. Red arrows highlight autophagic vesicles. **g** IHC analysis was used to compare protein expression levels of autophagy markers (p62 and LC3B) in Grade II and Grade V NP tissues. **h** Histological of IVDD model analysed by SO staining. **i**, **j** Representative images of immunofluorescence staining for p16^INK4a^ and LC3B protein in NP tissue at 2, 4 and 8 weeks after IVDD surgery. **k** Western blot analysis was conducted to evaluate the protein expression levels of p16^INK4a^, p21, p53, p62, and LC3B in both normal and TBHP-stimulated NPCs (*n* = 3). *P < 0.05, **P < 0.01. **l** Number of positive cells in normal and TBHP-stimulated NPCs detected by SA-β-gal. Data are expressed as mean ± SD

To investigate the role of NPCs senescence in the progression of IVDD, we obtained NP tissues from patients with different degrees of IVDD clinically (Fig. 1a). Compared with Grade II patients, the number of NPCs in the NP tissues of Grade V patients decreased significantly and the normal cell structure was lost (Fig. 1b), which is the manifestation of NPCs senescence. Subsequently, we detected the levels of typical biomarkers of senescent cells, p16^INK4a^ and p21, and found that with the aggravation of IVDD in patients, the levels of p16^INK4a^ and p21 in the NP tissues gradually increased (Fig. 1c, d). In addition, the primary NPCs isolated from the NP tissues of IVDD patients showed an increased positive rate of β-galactosidase staining and enhanced secretion of IL-1β (Fig. 1e, Fig. S1a, Fig. S4a). The subsequent results of transmission electron microscopy revealed that the accumulation of autophagic vesicles in IVDD-NPCs decreased, indicating autophagy deficiency in IVDD-NPCs (Fig. 1f). Therefore, we further measured the levels of autophagy markers LC3B and p62 in the NP tissues and found that the expression of LC3B was lower and the expression of p62 was higher in the NP tissues of IVDD patients (Fig. 1d, g and Fig. S1b).

To further explore the senescence of NPCs during the development of IVDD, a post-traumatic IVDD model was established by acupuncture surgery (Fig. S12a). In this model, with the progression of time, IVDD and Histological scores were significantly aggravated (Fig. 1h, Fig. S1c). The number of p16^INK4a^-positive NPCs was analysed and it was found that compared with the control group (sham-operated rats), in IVDD rats, these positive cells increased significantly over time in a time-dependent manner (Fig. 1i, Fig. S1f). And this increase was positively correlated with the deterioration of histological score. In addition, the primary NPCs isolated from the NP tissues of IVDD rats showed an increased positive rate of β-galactosidase staining (Fig. S1d). With the progression of IVDD, the expression level of LC3B in the intervertebral disc tissues of rats decreased significantly (Fig. 1j, Fig. S1g), and this decrease was negatively correlated with the histological score. The subsequent results of transmission electron microscopy revealed that the accumulation of autophagic vesicles in NPCs of IVDD rats decreased (Fig. S1e). These results indicate that NPCs senescence and impaired autophagy are tightly correlated with IVDD progression.

In vitro experiments, NPCs were treated with TBHP to simulate the degeneration process to further study the pathological mechanism of NPCs degeneration. NPCs treated with TBHP showed increased levels of senescence markers p16^INK4a^, p21, and p53, while the level of autophagy marker LC3B-II decreased and the level of p62 increased (Fig. 1k). The β-galactosidase assay also confirmed that TBHP promoted the senescence of NPCs (Fig. 1l, Fig. S1h). These data reveal that the senescence status of NPCs during IVDD is similar to that of NPCs in the in vitro degeneration model induced by TBHP.

### In vitro and vivo experiments SASP induces NPCs senescence and ECM degradation

**Fig. 2:**
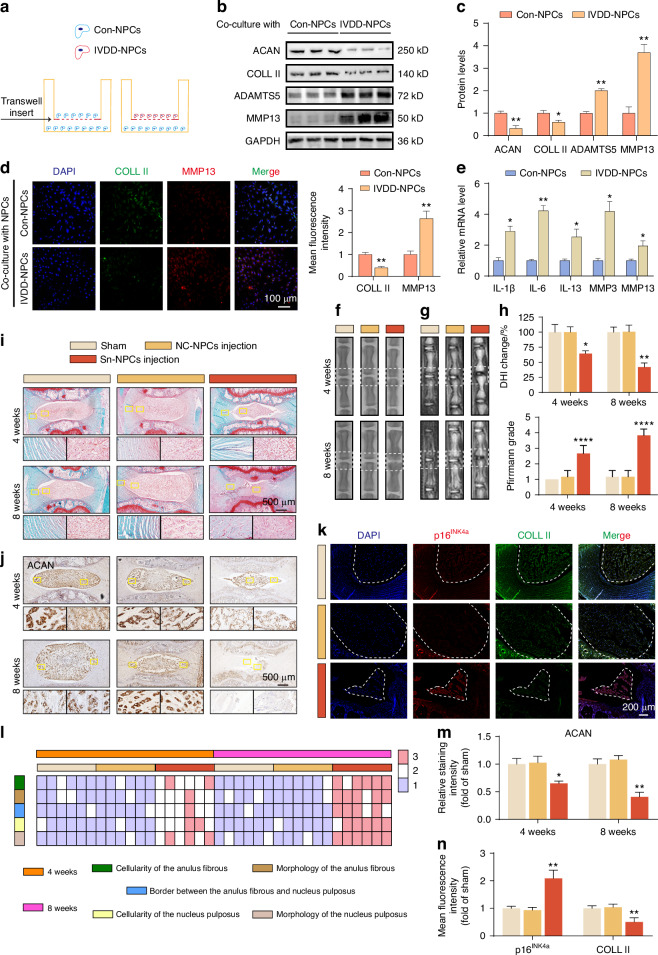
In vitro and vivo experiments SASP induces NPCs senescence and ECM degradation. **a** Diagram of the experimental design for the co-culture of human Con-NPCs or IVDD-NPCs with NPCs without direct contact. **b**, **c** Western blot analysis of ACAN, COLL II, ADAMTS5, and MMP13 protein expression levels in NPCs after 48 h of co-culture (*n* = 3). *P < 0.05, **P < 0.01. **d** Representative images of double immunostaining of COLL II and MMP13 proteins (*n* = 3). **e** qPCR detection of SASP-associated inflammatory cytokines (IL-1β, IL-6, and IL-13) and mRNA levels of MMP3 and MMP13 in Con-NPCs or IVDD-NPCs (*n* = 3). *P < 0.05, **P < 0.01. **f**, **g** Tail spine X-ray images and MRI images of rats from each group 4 and 8 weeks after injection. **h** DHI (%) of caudal spine X-ray images and Pfirrmann grading of MRI images of rats from each group (*n* = 6). *P < 0.05, **P < 0.01, ****P < 0.000 1. **i**, **l** Representative SO staining of caudal spine NP tissues from rats in each group after 4 and 8 weeks post-injection (*n* = 6); histological scores of rats from each group. **j**, **m** IHC analysis of ACAN protein expression levels in caudal spine NP tissues from rats in each group 4 and 8 weeks post-injection (*n* = 6). *P < 0.05, **P < 0.01. **k**, **n** Representative images of double immunostaining for p16^INK4a^ and COLL II proteins in caudal spine NP tissues from rats in each group after 8 weeks post-injection (*n* = 6). **P < 0.01. Data are expressed as mean ± SD

To further explore the cause of NPCs senescence during the progression of IVDD, we performed non-contact co-culture of primary NPCs from normal and IVDD patients (Fig. 2a). Interestingly, we detected that after Con-NPCs cells were co-cultured with IVDD-NPCs, the expression of senescence markers (p21, p16^INK4a^) and matrix degradation-related genes (MMP13, ADAMTS5) increased, while the expression of matrix synthesis-related genes (ACAN, COLL II) decreased (Fig. 2b–d). This indicates that Con-NPCs cells co-cultured with IVDD-NPCs undergo senescence and ECM degradation. Therefore, we speculate that IVDD-NPCs may promote the senescence and matrix degradation of NPCs by secreting certain substances. A previous study found that the SASP not only affects the cell microenvironment but also accelerates the senescence process of surrounding cells by promoting the production of inflammatory mediators such as inflammatory factors and matrix metalloproteinases (MMPs), which is similar to our research results. Therefore, we speculate that IVDD-NPCs promote the senescence and matrix degradation of Con-NPCs by secreting SASP. We performed qPCR on IVDD-NPCs and found that the levels of inflammatory factors (IL-1β, IL-6, IL-13) and matrix metalloproteinases (MMP3, MMP13) increased significantly (Fig. 2e). Therefore, we confirmed that during the progression of IVDD, NPCs secrete a large amount of SASP, leading to their senescence and matrix degradation.

To further study the role of SASP in the progression of IVDD in vivo, we used the DNA damage chemical bleomycin to induce strong senescence of rat NPCs (Fig. S2a), which is manifested by the induction of SA-β-Gal activity (Fig. S2b). Bleomycin-induced excessive senescence of NPCs secreted a large amount of SASP, which was further confirmed by the increase in SASP mRNA levels (Fig. S2c). Subsequently, 1.0 × 10^5^ normal and bleomycin-induced senescent NPCs were injected into the intervertebral discs of healthy rats (Fig. S2d). The examination results at 4 and 8 weeks after injection showed that compared with rats injected with normal NPCs, rats injected with senescent NPCs had significant loss of IVD, loss of NP tissue, disorder of annulus fibrosus (AF) tissue, and decreased ACAN expression (Fig. 2f–j). At the same time, the expression of p16^INK4a^ in the NP tissue increased and the expression of COLL II decreased (Fig. 2k–n). These results indicate that the abnormal secretion of SASP in NPCs mediates the progression of IVDD.

### In IVDD patients and surgically induced IVDD rat models, impaired autophagy is associated with the senescence of NPCs

**Fig. 3:**
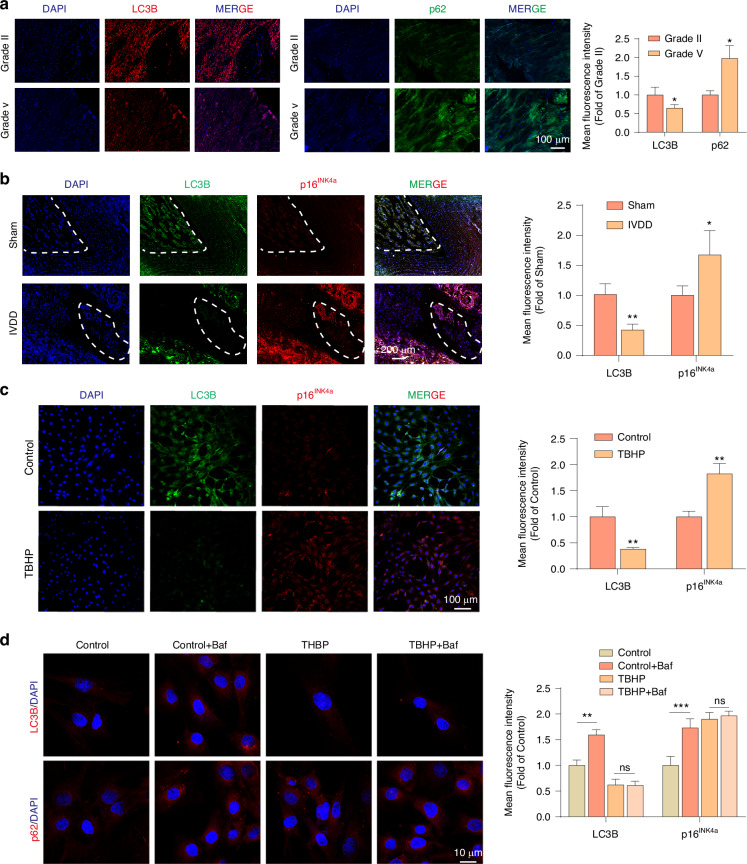
In IVDD patients and surgically induced IVDD rat models, impaired autophagy is associated with the senescence of NPCs. **a** Representative plots of double fluorescence immunostaining for the autophagy markers LC3B and p62 in NP tissues from patients with Grade II and Grade V degeneration (*n* = 6). *P < 0.05. **b** Representative plots of double fluorescence immunostaining for p16^INK4a^ and LC3B in NP tissues from rats 8 weeks post-IVDD surgery and sham-operated counterparts (*n* = 6). *P < 0.05, **P < 0.01. **c** Representative images of double fluorescence immunostaining for p16^INK4a^ and LC3B in normal and TBHP-induced senescent NPCs (*n* = 3). **P < 0.01. **d** Representative images of double fluorescence immunostaining for LC3B and p62 in normal and TBHP-induced senescent NPCs treated with bafilomycin A1 (Baf, 50 nmol·L^−1^) for 48 h (*n* = 3). ns > 0.05, **P < 0.01, ***P < 0.001. Data are expressed as mean ± SD

Recent studies suggest that impaired autophagy is involved in the ageing process across various organisms, potentially leading to premature cellular senescence.^31^ In this study, it was observed that in senescent NPCs of IVDD patients and IVDD model rats, the expression of autophagy marker LC3B-II decreased, while the level of p62 increased (Fig. 3a, Fig. S3a). In addition, in the surgically induced IVDD rat model and the TBHP-treated in vitro IVDD model, p16^INK4a^-positive cells showed a significant decrease in LC3B and a significant increase in p62 (Fig. 3b, c and Fig. S3b, c).

To verify the reduction of autophagic structures in senescent NPCs, the autophagy flux inhibitor Bafilomycin was used in this study. Bafilomycin can increase the level of LC3B by blocking lysosomal degradation. In the experiment, Bafilomycin treatment increased the levels of LC3B and p62 proteins in normal NPCs (Fig. 3d, Fig. S3d). However, the same treatment did not increase the expression of LC3B and p62 in senescent NPCs (Fig. 3d, Fig. S3d), indicating that the formation of autophagosomes in senescent NPCs is blocked and its lysosomal degradation function has been significantly weakened.

### Autophagy impairment in NPCs induces the secretion of SASP in a GATA4-dependent manner and accelerates cell senescence

**Fig. 4:**
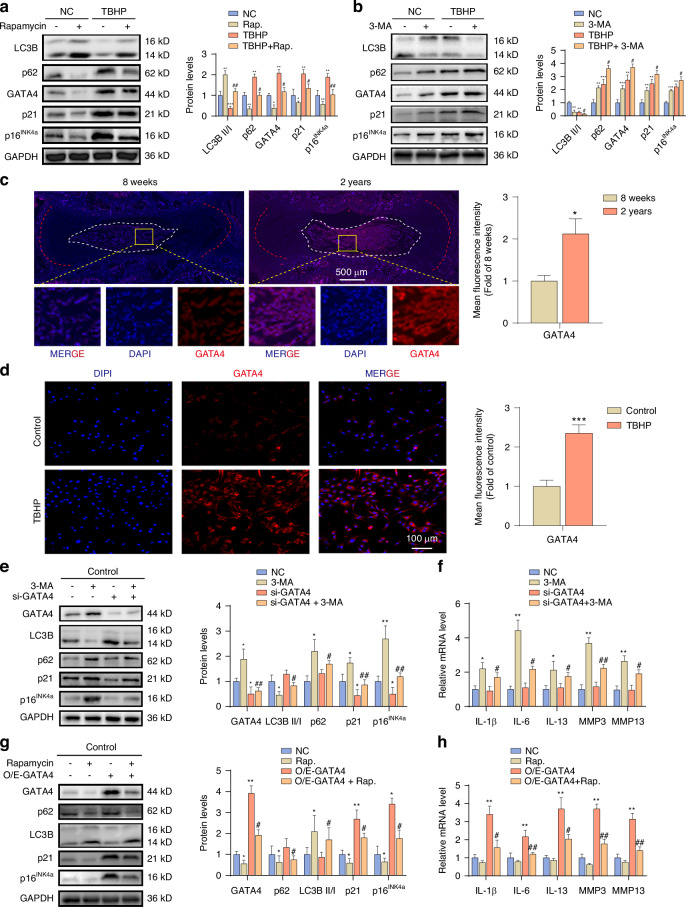
Autophagy impairment in NPCs induces the secretion of SASP in a GATA4-dependent manner and accelerates cell senescence. **a** Western blot analysis of protein levels of cellular autophagy markers (LC3B and p62), cellular senescence markers (p16^INK4a^ and p21), and GATA4 in normal NPCs and senescent NPCs after treatment with rapamycin (50 nmol·L^−1^) for 48 h (*n* = 3). *P < 0.05, **P < 0.01, ***P < 0.001, * VS NC; #*P* < 0.05, ##*P* < 0.01, # VS TBHP. **b** Western blot detection of LC3B, p62 in normal NPCs and senescent NPCs 48 h after transfection with 3-MA (1 mmol·L^−1^), protein levels of GATA4, p16^INK4a^ and p21 (*n* = 3). **P < 0.01, ***P < 0.001, * VS NC; #*P* < 0.05, # VS TBHP. **c** Representative images of immunofluorescence staining of GATA4 protein in NP tissues of aged rats (2 years) and control rats (8 weeks) (*n* = 6). **P < 0.01. **d** Representative images of fluorescence staining of GATA4 protein in normal NPCs and senescent NPCs induced by TBHP (*n* = 3). ***P < 0.01. **e** Western blot analysis of protein levels of LC3B, p62, GATA4, p16^INK4a^, and p21 in Con-NPCs after retargeting silencing of GATA4 after 48 h of treatment with 3-MA (*n* = 3). *P < 0.05, **P < 0.01, * VS NC; #*P* < 0.05, ##*P* < 0.01, # VS si-GATA4. **f** qPCR analysis of mRNA levels of SASP-associated inflammatory factors (IL-1β, IL-6, IL-13), MMP3, and MMP13 in the indicated groups (*n* = 3). *P < 0.05, *P < 0.05, **P < 0.05, * VS NC; #*P* < 0.05, ##*P* < 0.01, # VS si-GATA4. **g** Western blot detection of LC3B, p62 in Con-NPCs after treatment with rapamycin for 48 h and then targeted overexpression of GATA4, protein levels of GATA4, p16^INK4a^ and p21 (*n* = 3). *P < 0.05, **P < 0.01, * VS NC; #*P* < 0.05, # VS O/E-GATA4. **h** qPCR analysis of mRNA levels of SASP-associated inflammatory factors (IL-1β, IL-6, IL-13), MMP3, and MMP13 in the indicated groups (*n* = 3). **P < 0.01, * VS NC; #*P* < 0.05, ##*P* < 0.01, # VS O/E-GATA4. Data are expressed as mean ± SD

To assess the relationship between autophagy and NPCs senescence, we treated with the autophagy activator rapamycin and the autophagy inhibitor 3-methyladenine (3-MA). The results showed that rapamycin significantly increased the ratio of LC3B-II/I and inhibited the expression of p16^INK4a^, p21, p62, and IL-1β, suggesting that autophagy activation can inhibit NPCs senescence (Fig. 4a, Fig. S4b). While 3-MA decreased the LC3B-II/I ratio and increased the levels of p62, p16^INK4a^, p21, and IL-1β, indicating that inhibiting autophagy can promote senescence (Fig. 4b, Fig. S4b). This indicates that the decrease in autophagy activity is associated with NPCs senescence. Furthermore, we observed an increase in the senescence-associated transcription factor GATA4^32^ in both in vivo and ex vivo senescent NPCs (Fig. 4c, d), and knockdown of GATA4 decreased IL-1β secretion (Fig. S4c). Activation of autophagy decreased GATA4 expression, while inhibition of autophagy upregulated GATA4 (Fig. 4a, b), suggesting that autophagy may inhibit senescence by inhibiting GATA4 expression. To confirm this, we overexpressed or knocked down GATA4 in control cells and found that GATA4 knockdown alleviated the upregulation of p16^INK4a^, p21, and SASP induced by 3-MA (Fig. 4e, f), while GATA4 overexpression reversed the inhibitory effect of rapamycin on these indicators (Fig. 4g, h). This indicates that autophagy can affect the senescence process of NPCs by regulating GATA4.

In summary, the decrease in autophagy activity can promote the senescence of NPCs and SASP secretion through GATA4.

### Decreased expression of ATG4a in senescent NPCs

**Fig. 5:**
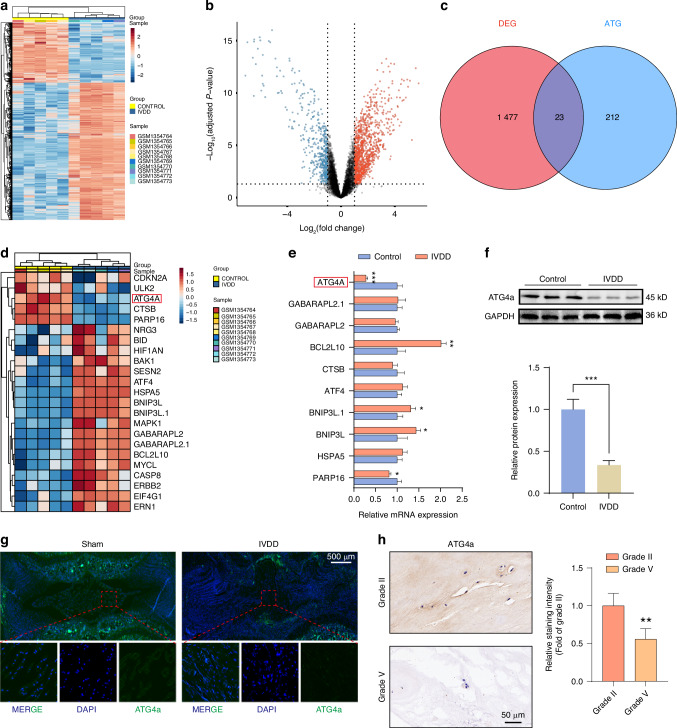
Decreased expression of ATG4a in senescent NPCs. **a**, **b** Heatmap and volcano plots illustrate the differential expression of genes (logFC < −1 or logFC > 1, *P* < 0.05) in NP tissues from normal subjects and IVDD patients (GSE56081). **c** An intersection plot of differentially expressed genes and autophagy-related genes is shown. **d** A heatmap analysis of differentially expressed autophagy-related genes in NP tissues from normal subjects and IVDD patients is displayed. **e** qPCR analysis quantified mRNA expression levels of the top 10 confidence genes in differential autophagy-related genes in NP tissues from normal subjects and IVDD patients (*n* = 3). **f** Western blot analysis of ATG4a protein levels in normal NPCs or IVDD-NPCs (*n* = 3). ***P < 0.001. **g** Representative images of fluorescence immunostaining of ATG4a protein in NP tissues from rats 8 weeks after IVDD surgery and sham-operated rats. **h** IHC analysis was used to compare protein expression levels of ATG4a in Grade II and Grade V NP tissues (*n* = 6). **P < 0.01. Data are expressed as mean ± SD

To further explore the molecular mechanism of autophagy impairment in senescent NPCs, we first analysed the transcriptome sequencing data of NP tissues from normal individuals and IVDD patients in the Gene Expression Omnibus database (GSE56081). We found 1 500 significantly differentially expressed mRNAs between the control group and the IVDD group (logFC < −1 or logFC > 1, *P* < 0.05). These differences were visualised by heatmap and volcano plot (Fig. 5a, b). Our preliminary experimental results show that the impaired autophagy of NPCs is significantly associated with the progression of IVDD. Therefore, we focused on the autophagy-related genes among the 1 500 differentially expressed genes. We identified 23 autophagy-related genes (Fig. 5c) and performed a heatmap analysis of these 23 common genes (Fig. 5d). We verified the 10 genes with the highest reliability among these 23 genes using qPCR, and the results showed that the expression of ATG4a, a key enzyme for autophagosome formation, decreased significantly in senescent NPCs (Fig. 5e). In addition, I verified the expression of ATG4a in human NP tissues, NP tissues of IVDD rats, and TBHP-induced senescent NPCs, and also found that its expression level decreased significantly in senescent NPCs (Fig. 5f–h, Fig. S5a–c).

### Deficiency of ATG4a mediates autophagy impairment in NPCs and accelerates NPCs senescence and IVDD through GATA4

**Fig. 6:**
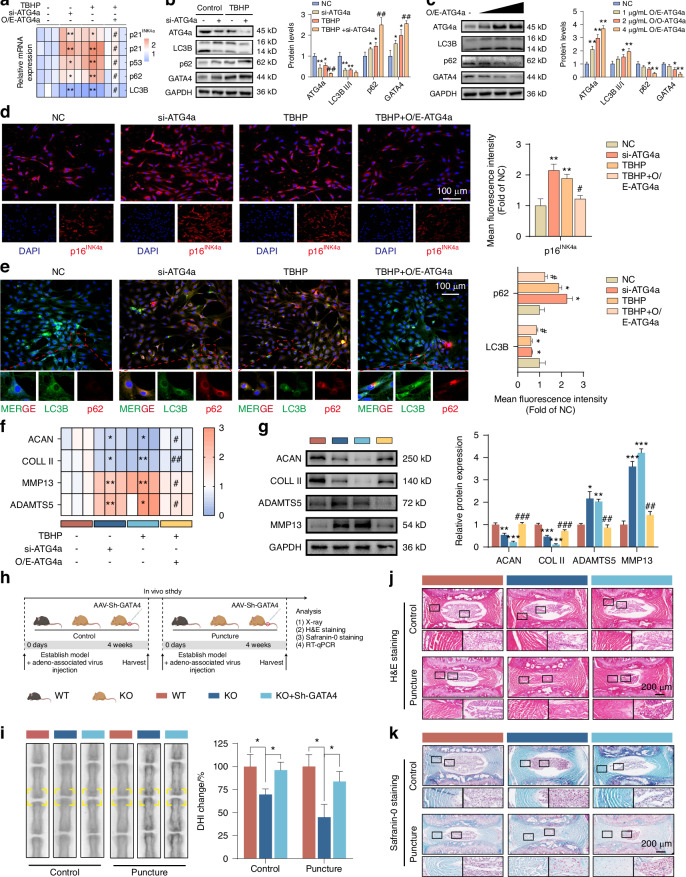
Deficiency of ATG4a mediates autophagy impairment in NPCs and accelerates NPCs senescence and IVDD through GATA4. **a** qPCR analysis quantified mRNA expression levels of autophagy markers (LC3B and p62) and cellular senescence markers (p16^INK4a^, p21, and p53) after silencing or overexpression of ATG4a in normal and TBHP-induced senescent NPCs (*n* = 3). *P < 0.05, **P < 0.01, * VS NC; #*P* < 0.05, # VS TBHP. **b** Western blot analysis measured the protein levels of ATG4a, LC3B, p62, and GATA4 after silencing of ATG4a in normal and TBHP-induced senescent NPCs (*n* = 3). *P < 0.05, **P < 0.01, * VS NC; ##*P* < 0.01, # VS TBHP. **c** Western blot analyses assessed protein levels of ATG4a, LC3B, p62, and GATA4 after transfection with O/E-ATG4a at different doses (1, 2, and 4 µg/mL) in normal NPCs (*n* = 3). *P < 0.05, **P < 0.01. **d** Immunofluorescence analysis provided representative images of p16^INK4a^ protein staining after silencing or overexpression of ATG4a in normal and senescent NPCs (*n* = 3). *P < 0.05, **P < 0.01, * VS NC; #*P* < 0.05, # VS TBHP. **e** Representative images from immunofluorescence analysis of dual fluorescence staining for LC3B and p62 proteins after silencing or overexpression of ATG4a in normal and senescent NPCs (*n* = 3). *P < 0.05, * VS NC; #*P* < 0.05, # VS TBHP. **f** qPCR analysis quantified mRNA expression levels of ACAN, COLL II, ADAMTS5, and MMP13 after silencing or overexpression of ATG4a in normal and TBHP-induced senescent NPCs (*n* = 3). *P < 0.05, **P < 0.01, * VS NC; #*P* < 0.05, # VS TBHP. **g** Western blot analysis of the protein expression levels of ACAN, COLL II, ADAMTS5, and MMP13 after silencing or overexpression of ATG4a in normal and TBHP-induced senescent NPCs (*n* = 3); *P < 0.05, **P < 0.01, ***P < 0.001, * VS NC; ##*P* < 0.01, ###*P* < 0.001, # VS TBHP. **h** Schematic illustration of IVDD model establishment and experiment design to evaluate the effects of ATG4a and GATA4 in vivo. **j** Radiographic presentation of control and IVDD model by X-ray (*n* = 6). *P < 0.05. **i**, **k** Histological of the IVDD model analysed by HE staining and SO staining (*n* = 6). Data are expressed as mean ± SD

Given that we found a significant decrease in the expression of ATG4a in senescent NPCs, we further verified whether autophagy impairment in senescent NPCs is related to the decreased expression of ATG4a. We found that knockout of ATG4a in NPCs led to a downregulation of the LC3B-II/I level and an upregulation of p62, p16^INK4a^, p21, p53, and GATA4 levels (Fig. 6a–e). Conversely, with the increase in the degree of ATG4a overexpression in NPCs, the LC3B-II/I level gradually increased, and the levels of p62, p16^INK4a^, p21, p53, and GATA4 gradually decreased, and the secretion of the inflammatory factor IL-1β was reduced (Fig. 6a–e, Fig. S4c). In the cell proliferation and senescence experiments, we found that the knockout of ATG4a promoted cell senescence, while overexpression of ATG4a contributed to cell proliferation (Fig. S6a, b). These results prove that the decrease in the level of ATG4a in NPCs can promote the expression of GATA4, ultimately leading to a decrease in its autophagy function and accelerating cell senescence.

In addition to NPCs, the ECM is another major component of intervertebral disc tissue. Therefore, we detected the expression of matrix synthesis-related genes (ACAN, COLL II) and degradation-related genes (MMP13, ADAMTS5). The results showed that in senescent NPCs, overexpression of ATG4a promoted the synthesis of the ECM and inhibited the degradation of the ECM (Fig. 6f, g and Fig. S6c, d). To further explore the key role of ATG4a in IVDD, we constructed ATG4a knockout mice using CRISPR/cas9-mediated genome editing technology (Fig. S7a) and verified its knockout efficiency and autophagy function in the intervertebral discs of mice by IHC staining (Fig. S7c, d), and the autophagy markers in the NP tissue of KO mice decreased significantly. To verify the role of the ATG4a/GATA4 axis in IVDD, we established a rat IVDD model and introduced the GATA4 adenovirus-associated virus (AAV) into NPCs through intradiscal injection (Fig. 6h). The effectiveness of virus infection was evaluated by qPCR of GATA4 in NP tissue (Fig. S7b). A prominent clinical manifestation of intervertebral disc degeneration is the presence of a pain phenotype. Behavioural experiments showed that KO mice showed significantly enhanced heat hyperalgesia and mechanical allodynia on the 7th and 14th days after surgery. In contrast, after injection of AAV-sh-GATA4, the pain sensation of mice was significantly reduced (Fig. S8a, b). Histological analysis of IVD showed that 2-month-old KO mice developed degeneration, manifested as the collapse of the IVD space, narrowing of the endplate height, and a moderate decrease in the number of NPCs (Fig. 6i–k, Fig. S8c). To further clarify the role of ATG4a in the development of IVDD, we established a 4-week surgically induced IVDD model in WT and KO mice (Fig. 6h). The degenerative changes of NPs in the IVD puncture group were obvious, while the degenerative changes of NPs and IVD in KO mice were more severe (Fig. 6i–k, Fig. S8c). These results were further confirmed in X-ray and histological evaluations, showing progressive narrowing of the IVD space and a significantly decreased histological score in the KO puncture group (Fig. 6i–k, Fig. S8c). To further verify the regulatory effect of ATG4a on GATA4 in vivo, we specifically silenced GATA4 in KO mice and detected the mRNA level of GATA4 in NPs (Fig. S7b) and found that the mRNA of GATA4 in KO mice increased significantly. And specific silencing of GATA4 in KO mice can reverse IVDD in KO mice (Fig. 6i–k, Fig. S8c).

In summary, our study found that the deficiency of ATG4a mediates the autophagy impairment of NPCs and accelerates NPCs senescence and IVDD through the GATA4-dependent pathway.

### METTL3 regulates ATG4a through m^6^A modification

**Fig. 7:**
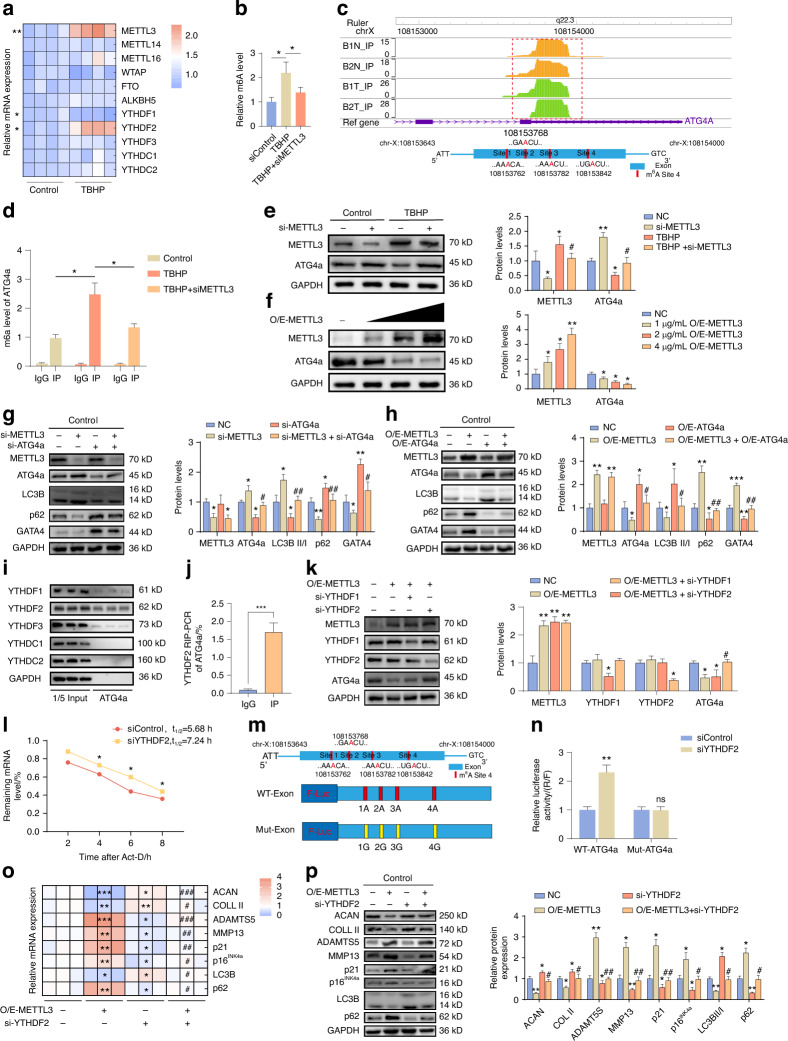
METTL3-mediated m^6^A modification induces attenuation of ATG4a transcripts in a YTHDF2-dependent manner. **a** qPCR analysis of mRNA levels of m^6^A-related proteins (*n* = 3). *P < 0.05, **P < 0.01. **b** Quantification of m6A modification level using ELISA-based m6A colourimetric assay (*n* = 3). *P < 0.05. **c** PEAK of ATG4a transcript by MeRIP-Seq (GSE169484) and m6A sites in ATG4a. **d** MeRIP-qPCR analysis of m^6^A modification level of ATG4a (*n* = 3). *P < 0.05. **e** Western blot analysis of ATG4a protein level after METTL3 knockdown (*n* = 3). *P < 0.05, **P < 0.01, * VS NC; #*P* < 0.05, # VS TBHP. **f** Western blot analysis of ATG4a protein level after overexpression of METTL3 (*n* = 3). *P < 0.05, **P < 0.01. **g** Western blot analysis of protein levels of METTL3, ATG4a, LC3B, p62 and GATA4 after silencing METTL3 and ATG4a (*n* = 3). *P < 0.05, **P < 0.01, * VS NC; #*P* < 0.05, ##*P* < 0.01, # VS si-ATG4a. **h** Western blot analyses of protein levels of METTL3, ATG4a, LC3B, p62 and GATA4 after overexpression of METTL3 and ATG4a (*n* = 3). *P < 0.05, **P < 0.01, ***P < 0.001, * VS NC; #*P* < 0.05, ##*P* < 0.01, # VS O/E-ATG4a. **i** RNA pull-down followed by western blot analysis of the isolated proteins of stability-associated readers Western blot. **j** Confirmation of the recognition of YTHDF2 with ATG4a mRNA by RIP-qPCR; ***P < 0.001. **k** Western blot analysis of overexpression of protein levels of METTL3, YTHDF1, YTHDF2, and ATG4a in cells after continued silencing of YTHDF1 or YTHDF2 after METTL3 (*n* = 3). *P < 0.05, **P < 0.01, * VS NC; #*P* < 0.05, # VS O/E-METTL3. **l** mRNA stability assay of ATG4a in NPCs with or without YTHDF2 silencing (*n* = 3). *P < 0.05. **m** All m6A sites mutation (Mut) of ATG4a. **n** Dual-luciferase reporter assay of wild-type or site-mutant NPCs with or without YTHDF2 silencing (*n* = 3). ns >0.05, *P < 0.05. **o** qPCR analysis of mRNA levels of overexpression of METTL3 followed by continued silencing of YTHDF2, ACAN, COLL II, ADAMTS5, MMP13, p21, p16^INK4a^, LC3B and p62 (*n* = 3). *P < 0.05, **P < 0.01, ***P < 0.001, * VS NC; #*P* < 0.05, ##*P* < 0.01, ###*P* < 0.001, # VS O/E-METTL3. **p** Western blot analysis of overexpression of METTL3 followed by continued silencing of YTHDF2, ACAN, COLL II, ADAMTS5, MMP13, p21, p16^INK4a^, LC3B and p62 protein levels (*n* = 3). *P < 0.05, **P < 0.01, * VS NC; #*P* < 0.05, ##*P* < 0.01, # VS O/E-METTL3. Data are expressed as mean ± SD

Recent studies have increasingly highlighted the crucial role of m^6^A modification in various physiological processes, particularly in the regulation of autophagic activity.^20,33^ To study the role of m^6^A modification in IVDD degeneration and autophagy of NPCs, we detected the expression levels of methyltransferases and demethylases (the main regulators of m6A modification) in NPCs by Western blotting and qPCR and found that the expression level of the m6A methyltransferase METTL3 increased in senescent NPCs (Fig. 7a, Fig. S9a–e). Furthermore, the m^6^A colourimetric assay based on Elisa indicated that although TBHP treatment could increase the m^6^A modification level in NPCs, knockdown of METTL3 could significantly reduce the m^6^A modification level (Fig. 7b). To study the role of m^6^A modification in regulating ATG4a, we analysed the MeRIP-seq data (GSE169484). We found that the m^6^A peak of ATG4a was enriched in the exon region from 108153643 to 108154000. Combined with the online bioinformatics tool SRAMP^34^ (http://www.cuilab.cn/sramp/), we identified 4 highly reliable m^6^A binding sites within the exon region of the ATG4a transcript (Fig. 7c). In addition, the methylation levels of these 4 adenine sites differed significantly. Through m^6^A-RNA immunoprecipitation quantitative PCR (MeRIP-qPCR) analysis, it was found that the m^6^A level of ATG4a in senescent NPCs was significantly higher than that in normal NPCs, and silencing METTL3 could significantly reduce the m^6^A level of ATG4a (Fig. 7d). This suggests that METTL3 regulates m^6^A modification of ATG4a during NPCs senescence. To further study the effect of METTL3 on the expression of ATG4a, we constructed in vitro models of METTL3 overexpression and silencing. The results showed that knockdown of METTL3 could increase the expression of ATG4a in senescent NPCs and reduce the secretion of IL-1β (Fig. 7e, Fig. S4c). In contrast, overexpression of METTL3 significantly reduced the expression of ATG4a (Fig. 7f). Considering that we observed a negative correlation between METTL3 and ATG4a expression and its effect on IL-1β secretion, we further verified whether METTL3 affects cell autophagy and ageing by regulating the expression of ATG4a. Knockdown of ATG4a reversed the increase in LC3B-II level induced by METTL3 silencing and increased the expression of p62 and GATA4 in Con-NPCs (Fig. 7g). While overexpression of ATG4a effectively alleviated the decrease in LC3B-II level induced by METTL3 overexpression and reduced the expression of p62 and GATA4 in Con-NPCs (Fig. 7h).

These results suggest that the autophagy defect in senescent NPCs may be caused by METTL3-mediated inhibition of ATG4a expression.

### m^6^A modification induces the attenuation of ATG4a transcript in a YTHDF2-dependent manner

Although METTL3 is the “writer” of m^6^A on ATG4a, the potential m6A - selective binding proteins need to recognise m6A - modified mRNA and exert regulatory functions. We found that the expression of YTHDF1 decreased and the expression of YTHDF2 increased in senescent NPCs (Fig. 7a, Fig. S9a). To unravel the molecular mechanism by which m^6^A modification regulates the expression of ATG4a, RNA pull-down was performed, and then Western blotting analysis was performed on the proteins isolated from senescent NPCs. The results showed that YTHDF2 had a stronger interaction with ATG4a mRNA, and additional RIP-qPCR analysis further revealed this understanding (Fig. 7i, j). To further clarify whether YTHDF1 or YTHDF2 selectively targets m^6^A-modified mRNA of ATG4a to regulate its expression in NPCs, we transfected NPCs with METTL3 plasmid and then treated them with or without si-YTHDF1 and si-YTHDF2, respectively. We found that silencing YTHDF2 could significantly alleviate the decrease in ATG4a protein expression induced by METTL3, while silencing YTHDF1 did not affect ATG4a protein expression (Fig. 7k). Then we constructed an overexpression and silencing system of YTHDF2 in vitro and obtained that YTHDF2 silencing could upregulate the expression of ATG4a in senescent NPCs and reduce the secretion of IL-1β (Figs. S10a and S4c). In contrast, upregulation of YTHDF2 could significantly reduce the expression of ATG4a (Fig. S10b). These experiments indicate that the m^6^A modification of ATG4a affects its expression through YTHDF2. To further explore the mechanism by which YTHDF2 regulates the expression of ATG4a, we first treated NPCs with cycloheximide (CHX) to block translation. The Western blotting results confirmed that the half-life of ATG4a protein was similar, indicating that m6A-related ATG4a expression was not related to protein stability (Fig. S10e). Next, we treated NPCs with Act-D to block transcription and used qPCR to detect the half-life of pre-RNA (precursor) and mat-RNA (mature) at multiple points. The stability of ATG4a mature mRNA in YTHDF2 - knockdown NPCs increased, and the half-life was prolonged (Fig. 7l). Next, we performed a dual-luciferase reporter assay, and the results showed that the change in YTHDF2 expression had no effect on the mutant ATG4a (Fig. 7m, n). These data indicate that the decrease in ATG4a stability caused by m^6^A modification is mediated by YTHDF2.

Knockdown of ATG4a reversed the increase in LC3B-II level induced by YTHDF2 silencing and increased the expression of p62 and GATA4 in Con-NPCs (Fig. S10f). While overexpression of ATG4a effectively alleviated the decrease in LC3B-II level induced by YTHDF2 overexpression and reduced the expression of p62 and GATA4 in Con-NPCs (Fig. S10g). These results suggest that the autophagy defect in NPCs may be due to YTHDF2-induced inhibition of ATG4a expression. In addition, the ageing and degeneration of human NPCs caused by high m^6^A modification of ATG4a induced by overexpression of METTL3 can be eliminated by silencing YTHDF2 (Fig. 7o, p, Fig. S10h).

In summary, our research results indicate that METTL3 regulates the stability of ATG4a mRNA in a YTHDF2-dependent manner to affect autophagy damage and senescence in NPCs.

### METTL3 upregulation is induced by epigenetic alterations in H3K4me3

**Fig. 8:**
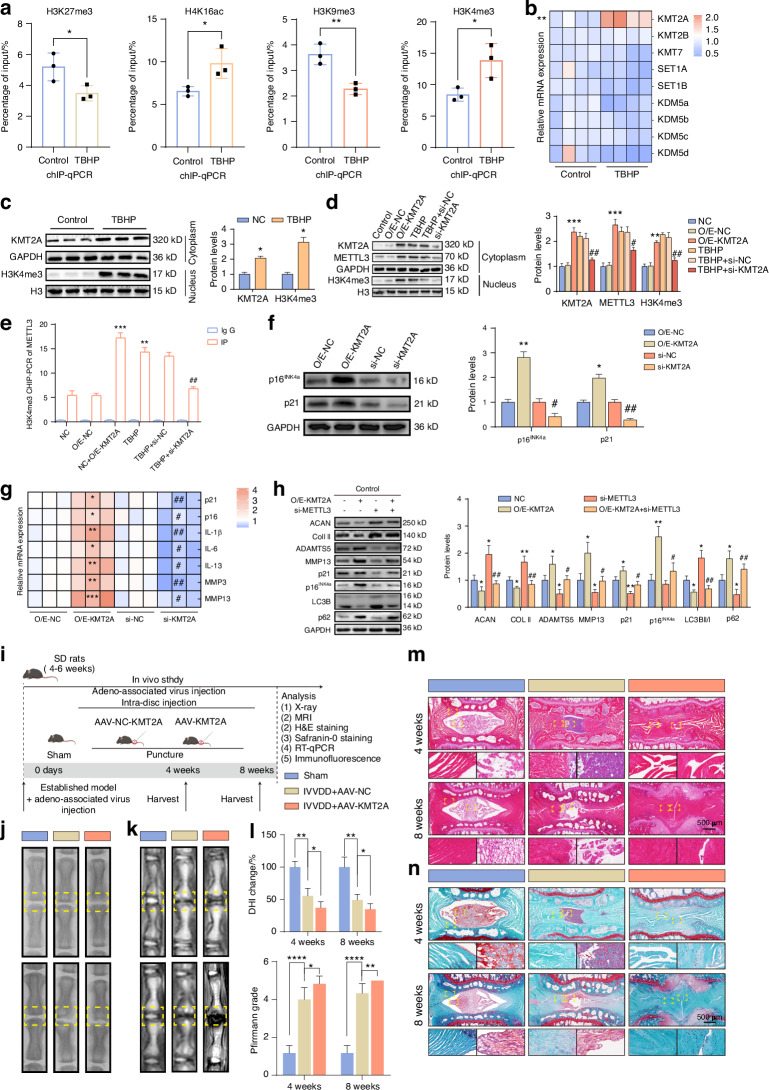
METTL3 upregulation is induced by epigenetic alterations in H3K4me3. **a** ChIP-qPCR analysis of H3K27me3, H4K16ac, H3K9me3, H3K4me3 modifications of METTL3 promoter (*n* = 3). *P < 0.05, **P < 0.01. **b** qPCR analysis of methyltransferase and demethylase transcriptome levels in normal NPCs and senescent NPCs induced by TBHP (*n* = 4). **P < 0.01. **c** Western blot analysis of KMT2A and H3K4me3 protein levels in normal NPCs and senescent NPCs induced by TBHP (*n* = 3). *P < 0.05. **d** Western blot detection of protein levels of KMT2A, METTL3 and H3Keme3 after overexpression of KMT2A or silencing of KMT2A in normal NPCs and senescent NPCs induced by TBHP (*n* = 3). **P < 0.01, ***P < 0.001, * VS NC; #*P* < 0.05, ##*P* < 0.01, # VS TBHP. **e** ChIP-qPCR analyses of H3K4me3 modification in the METTL3 promoter after overexpression of KMT2A or silencing of KMT2A in normal NPCs and in senescent NPCs induced by TBHP (*n* = 3). **P < 0.01, ***P < 0.001, * VS NC; ###*P* < 0.01, # VS TBHP. **f** Western blot detection of protein levels of p16^INK4a^ and p21 after overexpression or silencing of KMT2A in normal NPCs (*n* = 3). *P < 0.05, **P < 0.01, * VS O/E-NC; #*P* < 0.05, ##*P* < 0.01, # VS si-NC. **g** qPCR analyses of mRNA levels of SASP-associated inflammatory factors (IL-1β, IL-6, IL-13), MMP3, MMP13, after overexpression or silencing of KMT2A in normal NPCs (*n* = 3). *P < 0.05, **P < 0.01, ***P < 0.001, * VS O/E-NC; #*P* < 0.05, ##*P* < 0.01, # VS si-NC. **h** Western blot assay for overexpression of KMT2A in Con-NPCs followed by continued silencing of METTL3 for each of the indicated genes (ACAN, COLL II, ADAMTS5, MMP13, p21, p16^INK4a^, LC3B and p62) at the protein level (*n* = 3). *P < 0.05, **P < 0.01, * VS NC; #*P* < 0.05, ##*P* < 0.01, # VS si-METTL3. **i** Schematic illustration of IVDD model establishment and experiment design to evaluate the effects of KMT2A in vivo. **j**–**l** Radiographic presentation of control and IVDD model by X-ray and MRI (*n* = 6). *P < 0.05, **P < 0.01, ***P < 0.001, ****P < 0.000 1. **m**, **n** Histological of IVDD model analysed by HE staining and SO staining. Data are expressed as mean ± SD

Histone modification, a key epigenetic process, regulates gene expression by altering chromatin accessibility and plays a significant role in cellular senescence.^35^ Age-related epigenetic alterations, such as increases in H4K16ac and H3K4me3, alongside decreases in H3K9me3 and H3K27me3, have been characterised as markers of ageing.^36^ To ascertain whether these epigenetic marks could regulate METTL3 expression, we initially examined the METTL3 promoter’s modifications via the WashU Epigenome Browser^37^ (https://epigenomegateway.wustl.edu/) and found abundant signals in the promoter region of METTL3 (Fig. S11a), indicating that METTL3 is regulated by histone modification. Moreover, chromatin immunoprecipitation (ChIP) assays were used to measure the levels of these epigenetic modifications. The ChIP-qPCR results showed that the increase in histone H3 lysine 4 (H3K4) trimethylation (H3K4me3) was the most significant in senescent NPCs (Fig. 8a). To clarify its specific mechanism, we analysed the expression of lysine methyltransferase and demethylase of H3K4me3 and found that the expression of methyltransferase KMT2A increased most significantly in senescent NPCs (Fig. 8b, c). In normal NPCs, overexpression of methyltransferase KMT2A could upregulate the H3K4me3 modification and promote the expression of METTL3; while silencing methyltransferase KMT2A in senescent NPCs led to the opposite result (Fig. 8d, e).

To further understand the functional role of KMT2A in regulating the ageing of NPCs, we overexpressed or knocked out KMT2A in normal NPCs. We demonstrated that the upregulation of KMT2A significantly increased the expression of p16^INK4a^ and p21 in NPCs (Fig. 8f) and the mRNA level of SASP (Fig. 8g). The β-galactosidase assay also confirmed that KMT2A promotes the ageing of NPCs (Fig. S11b). Knockout of KMT2A inhibited the senescence in NPCs and the secretion of IL-1β (Fig. 8f, g, Fig. S4c). In addition, the ageing and degeneration of NPCs in human NPCs induced by overexpression of methyltransferase KMT2A and high modification of H3K4me3 can be eliminated by silencing METTL3-mediated m6A methylation reduction (Fig. 8h). To study the role of KMT2A in IVDD, we established a rat IVDD model and introduced the KMT2A AAV into NPCs through intradiscal injection (Fig. 8i). The effectiveness of virus infection was evaluated by IHC detection (Fig. S11c). The degenerative changes of NPs in the IVD puncture group were obvious, and the degenerative changes of NPs and IVD after overexpression of KMT2A were more severe (Fig. 8j–n, Fig. S11d). These results were further confirmed in X-ray and histological evaluations. After overexpression of KMT2A, the IVD space showed progressive narrowing and the histological score significantly decreased (Fig. 8j–n, Fig. S11d). To further verify the regulatory effect of KMT2A on METTL3 in vivo, the expression of METTL3 in NPs was detected, and it was found that KMT2A promoted the expression of METTL3 (Fig. S11d).

Taken together, the above data further confirm that KMT2A upregulates METTL3 via H3K4me3 and promotes senescence in NPCs and IVDD progression.

### METTL3/ATG4a axis regulates the progression of IVDD in vivo

**Fig. 9:**
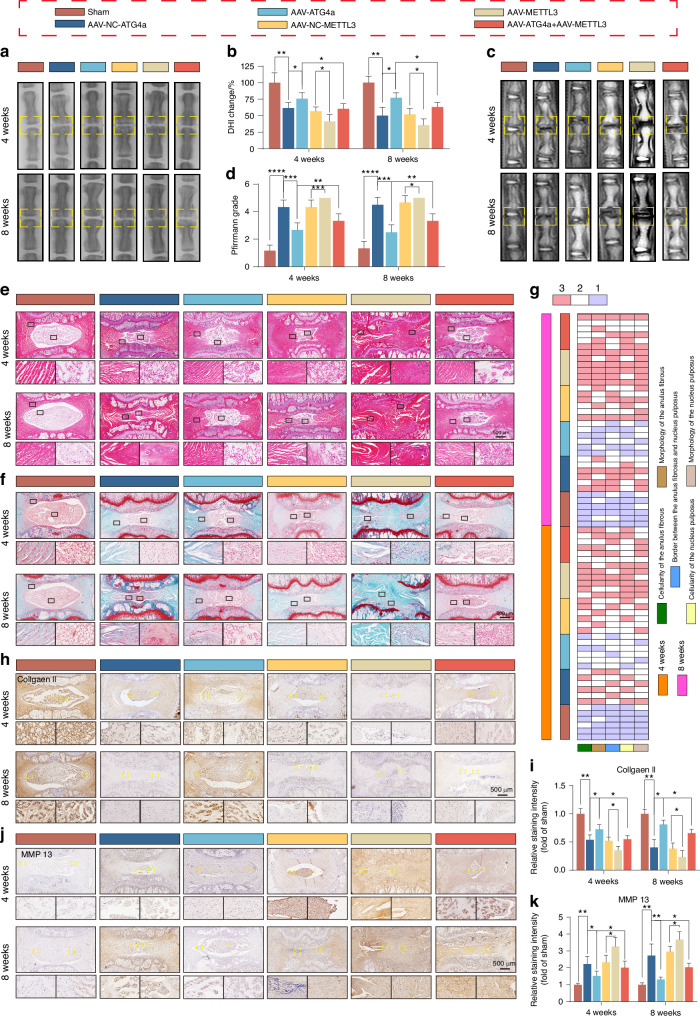
METTL3/ATG4a axis regulates the progression of IVDD in vivo. **a**, **b** Tail spine x-ray images and DHI changes in rats in each group at 4 and 8 weeks postoperatively (*n* = 6). *P < 0.05, **P < 0.01. **c**, **d** MRI images of the tail spine and changes in Pfirrmann score in rats in each group at 4 and 8 weeks postoperatively (*n* = 6). *P < 0.05, **P < 0.01, ***P < 0.001 ****P < 0.000 1. **e**–**g** HE and SO images of NP tissues of rat caudal spine in each group at 4 and 8 weeks postoperatively (*n* = 6); histological scores of rat NP tissues at 4 and 8 weeks postoperatively. **h**–**k** IHC detection of COLL II and MMP13 protein levels in rat tailbone NP tissues at 4 and 8 weeks after surgery (*n* = 6). *P < 0.05, **P < 0.01. Data are expressed as mean ± SD

To study the role of the METTL3/ATG4a axis in IVDD, we established a rat IVDD model and introduced ATG4a or METTL3 AAV into NPCs through intradiscal injection (Fig. S12a, b). The effectiveness of virus infection was evaluated by qPCR and IHC detection of ATG4a (Fig. S12c–e). Behavioural experiments showed that rats showed significantly enhanced heat hyperalgesia and mechanical allodynia on the 7th and 14th days after IVDD surgery. After overexpression of ATG4a, the pain sensation of mice was significantly reduced, while overexpression of METTL3 would aggravate heat hyperalgesia and mechanical allodynia, and overexpression of METTL3 could reverse the therapeutic effect brought by ATG4a (Fig. S13a, b). Through X-ray and MRI analysis, we found that after IVDD surgery, the height of the intervertebral space was significantly lost. In contrast, the AAV-ATG4a group significantly reversed this change. Notably, co-transfection of ATG4a and METTL3 counteracted the therapeutic effect brought by ATG4a overexpression and inhibited the expression of ATG4a mRNA in NP (Fig. 9a–d, Fig. S12c). In addition, we used histological staining techniques, namely H&E, S&O and COL II, MMP13, and LC3B immunohistochemical staining on intervertebral disc slices of different groups of rats. The results showed that overexpression of ATG4a increased the synthesis of ECM in NP, reduced degradation, activated autophagy function, and reduced the degree of IVDD (Fig. 9e–k, Fig. S13c). In contrast, overexpression of METTL3 on the basis of ATG4a overexpression showed the ability to counteract this effect (Fig. 9e–k, Fig. S13c).

In summary, these results indicate that METTL3 promotes the progression of IVDD by inhibiting the expression of ATG4a.

### METTL3/ATG4a axis regulates senescence of NPCs in vivo

**Fig. 10:**
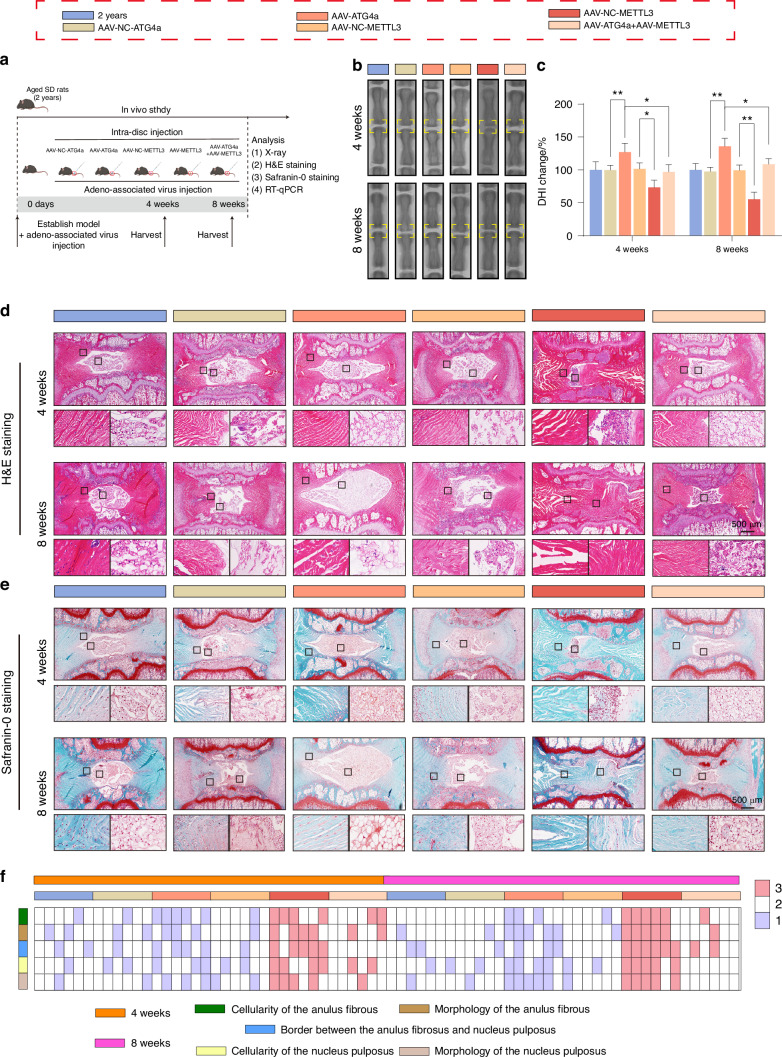
METTL3/ATG4a axis regulates senescence of NPCs in vivo. **a** The diagram for experimental design. **b**, **c** Changes in x-ray images and DHI of the tail spine of rats in each group after 4 and 8 weeks of injection (*n* = 6). *P < 0.05, **P < 0.01. **d**, **e** HE and SO staining images of IVD in the caudal spine of rats in each group 4 and 8 weeks after the injection procedure (*n* = 6). **f** Heat maps of histological scores 4 and 8 weeks after injection. Data are expressed as mean ± SD

To further investigate the role of the METTL3/ATG4a axis in the regulation of senescence in NPCs, we constructed a naturally ageing rat model and introduced ATG4a or METTL3 AAV into the intervertebral discs of ageing rats through intradiscal injection (Fig. 10a). Through X-ray analysis, we found that in ageing rats, the height of the IVD space was significantly lost. In contrast, transfection of ATG4a significantly reversed this change. Notably, co-transfection of ATG4a and METTL3 counteracted the therapeutic effect brought by ATG4a overexpression (Fig. 10b, c). In addition, we used histological staining techniques, especially H&E and S&O staining, to stain the intervertebral disc slices of different groups of rats. The results showed that the NP tissue structure of ageing rats disappeared, and the AF tissue structure was disordered. Overexpression of ATG4a could significantly improve the pathological changes of these tissues (Fig. 10d–f). Similarly, continuous overexpression of METTL3 could reverse the improvement of tissue pathology caused by the increase in ATG4a (Fig. 10d–f). Next, by detecting the expression levels of p16^INK4A^ and p21 to determine the ageing degree of NPCs in the intervertebral discs of each group of rats. Compared with the control group, the number of senescent NPCs in the NP tissue of rats injected with ATG4a AAV was significantly reduced (Fig. S14a–c). In addition, simultaneous overexpression of ATG4a and METTL3 AAV could reverse this effect (Fig. S14a–c).

In summary, these findings indicate that METTL3 promotes the senescent phenotype of NPCs and accelerates the progression of IVDD by inhibiting the expression of ATG4a.

## Discussion

**Fig. 11:**
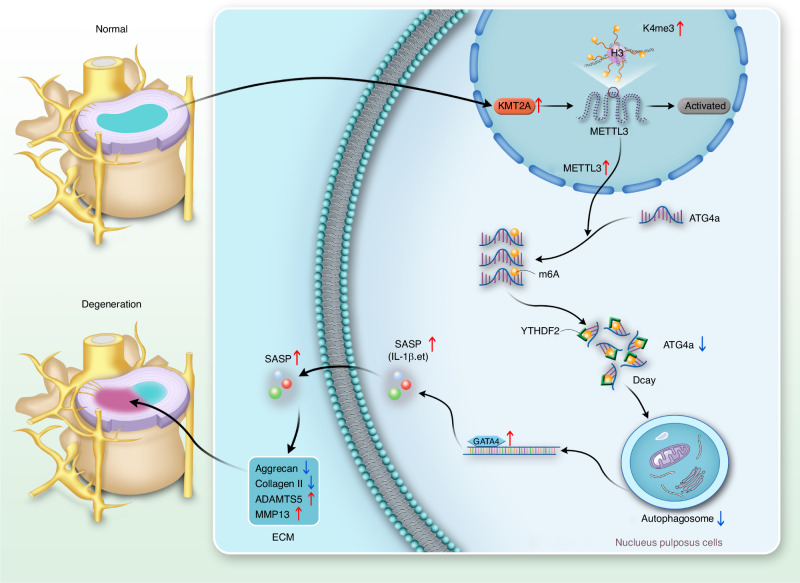
Schematic representation of the mechanisms leading to NPCs senescence and disc degeneration

Cellular senescence is increasingly recognised as a significant aetiological factor in IVDD, with evidence suggesting that the removal of senescent cells locally can decelerate the progression of IVDD.^38–40^ Recent research indicates that m^6^A modifications play roles in multiple musculoskeletal disorders.^41–43^ Our current study associates impaired autophagy during IVDD and ageing of NPCs with reduced ATG4a expression. We demonstrate that ATG4a overexpression can effectively mitigate impaired autophagy and ageing in NPCs. We discovered that the downregulation of ATG4a leads to increased GATA4 expression, stemming from an elevated SASP, which in turn promotes senescence and degeneration in otherwise normal NPCs. Further investigation revealed that METTL3-mediated m^6^A modification of ATG4a influences its mRNA stability in an m^6^A-YTHDF2-dependent manner. Moreover, increased METTL3 expression was found to be regulated via the H3K4me3 modification at the promoter, which is mediated by KMT2A (Fig. 11).

The production of pro-inflammatory and matrix-degrading molecules, collectively known as SASP, contributes to a chronic inflammatory microenvironment—a pivotal mechanism implicated in disc degeneration.^44^ Autophagy facilitates cell survival under stress by delivering dysfunctional organelles and macromolecules to lysosomes.^11^ Impaired autophagy is implicated in the pathogenesis of several age-related diseases.^45,46^ With age, autophagy efficiency declines, resulting in increased NPCs senescence and exacerbating IVDD severity. Thus, enhancing homoeostatic mechanisms in vivo has been proposed as an innovative approach to delay disc degeneration and lower IVDD risk.^47,48^ We found that ATG4a expression reduction, essential for autophagic vesicle formation and fusion,^49^ mediates impaired autophagy in IVDD-NPCs, while ATG4a overexpression activates autophagy, which in turn inhibits NPCs senescence. Additionally, our data indicate that the novel senescence regulator GATA4^32^ is significantly upregulated in IVDD-NPCs and that its elevation induces SASP and cellular senescence marker expression. Our findings suggest that autophagy regulates NPCs senescence in a GATA4-dependent manner and that modulating autophagy may offer a viable strategy for IVDD treatment.

m^6^A modification is one of the most prevalent RNA modifications, accounting for over 80% of all RNA methylation.^50^ Past studies have established the vital role of m^6^A modification in various physiological processes, including cellular senescence, tumour invasion, and cell differentiation.^51–53^ It has been reported that m^6^A-modified mRNA transcripts are less stable due to YTHDF2-mediated decay.^54^ In our study, we observed increased m^6^A modification of ATG4a mRNA in IVDD-NPCs, accompanied by a rise in METTL3 expression, a member of the methyltransferase complex, which is likely to enhance m^6^A levels.^55^ Furthermore, we showed that YTHDF2 regulates METTL3-mediated m^6^A modification of ATG4a. Our in vivo and in vitro experiments demonstrate that METTL3 deletion in IVDD-NPCs restores autophagy and decreases GATA4 expression, effectively reducing senescent cell accumulation in the NPCs and inhibiting IVDD progression.

Recent studies have only begun to explore the regulation of senescence in NPCs at the epitranscriptional level. In our study, we have discovered that METTL3, a key component of the methyltransferase complex, is significantly upregulated during NPCs senescence, which leads to increased formation of the methyltransferase complex. It is documented that enzymes like ALKBH5 and METTL3 can modulate tumour progression through epigenetic alterations of their promoters.^56^ Among the spectrum of epigenetic cell modifications, histone modifications are known to influence gene expression by altering chromatin accessibility and have been identified as a crucial element in cellular senescence.^35^ Notably, the increase in H4K16ac and H3K4me3 modifications, along with the decrease in H3K9me3 and H3K27me3 modifications, are characteristic epigenetic hallmarks of cellular senescence.^36^ Following up on these findings, we probed whether the upregulation of METTL3 in NPCs could be attributed to histone modifications. We found that the H3K4me3 modification of the METTL3 promoter, mediated by KMT2A, enhances its transcription, which in turn facilitates ATG4a m^6^A modification. The knockdown of KMT2A in cells significantly decreased METTL3 expression, augmented the expression level of ATG4a in NPCs, restored autophagy, and diminished GATA4 expression. Such changes effectively reduced the prevalence of senescent cells within the nucleus pulposus and impeded the advancement of IVDD.

In summary, the findings of this research enrich our understanding of the role METTL3 plays in the regulation of senescence and the progression of IVDD in NPCs through m^6^A modification. These insights reveal that disrupting ATG4a m^6^A modification or targeting the METTL3 /ATG4a /GATA4 axis can mitigate the progression of IVDD. This study also offers a potential epigenetic therapeutic strategy for the treatment of IVDD.

## Materials and methods

### Immunofluorescence staining and confocal laser scanning

Slides were rinsed with PBS, fixed in 4% paraformaldehyde for 15 min, permeabilized with 0.1% Triton X-100 for 5 min, and blocked with 10% goat serum for 1 h. Primary antibodies were applied at 1:400 overnight at 4 °C (refer to Table S1 for antibody specifics). Following three PBS washes, secondary antibodies and DAPI (Solarbio, C0065) staining were applied. Visualisation was done using a Zeiss LSM880 confocal (Germany).

### MeRIP-qPCR

The m^6^A immunoprecipitation (MeRIP) process was carried out using the Magna MeRIP™ m^6^A Kit (Merck Millipore, USA) in accordance with the manufacturer’s guidelines. Firstly, mRNA was isolated and treated with DNase I, then the mRNA was fragmented using RNA fragmentation reagents at 94 °C. After fragmentation, a termination buffer was added and the fragmented mRNA was collected by standard ethanol precipitation. Subsequently, 12 μg of anti-m^6^A antibody was pre-bound with 50 μL of microspheres in IP buffer at room temperature for 1 h. Then, 6 μg of fragmented mRNA was added to the antibody-microsphere mixture and incubated on a rotator at 4 °C for 4 h. After thorough washing, the immunoprecipitated complexes were digested with high-concentration protease K, the bound RNA was extracted using the phenol-chloroform method and then precipitated with ethanol for qPCR analysis. We evaluated the m6A modification in ATG4a by qPCR using specific primers (Table S3).

### ChIP-qPCR

NPCs chromatin immunoprecipitation (ChIP) was conducted per Abcam’s modified crosslinking ChIP protocol. Cells were fixed with 1% formaldehyde for 10 min at room temperature, sonicated, and the resultant chromatin incubated with primary antibodies (see Table S1) or control IgG (Abcam, USA). Protein G (Millipore, USA) precipitated the chromatin-antibody complexes. DNA was then extracted, and purified with Qiagen’s QIAquick PCR kit (China), and qPCR analysed using specific primers, with normalisation to input DNA. Data were derived from three separate experiments, with ChIP-qPCR primers detailed in Table S3.

### Animal model

Adult male Sprague-Dawley rats, aged 6 weeks and 24 months, were obtained from the Zhejiang Provincial Laboratory Animal Center, with approval from Wenzhou Medical University’s Laboratory Animal Ethics Committee. The caudal disc (Co 7/8) was identified by palpation and confirmed via radiographic vertebral count from the sacral region. A 27G needle was used to puncture the AF and inject adeno-associated virus (AAV) with specific sequences into the NP. Post-procedure, the rats’ health was monitored daily, and they were allowed unrestricted movement, regular feeding, and free access to water. This study protocol was approved by the Ethics Committee of Wenzhou Research Institute of State Science and Technology (No. WIUCAS23020208).

### Histological analysis

Rats were euthanized at 4 and 8 weeks post-surgery, and their intervertebral discs were harvested. The collected disc samples were fixed in 4% paraformaldehyde for one day and decalcified in 10% EDTA for one month. The decalcified samples were then embedded in paraffin and sectioned into 5 μm slices, inclusive of the endplates, annulus fibrosus, and nucleus pulposus. The structural changes in the intervertebral discs were examined using Hematoxylin and Eosin (H&E), Safranin-O/Fast Green (S&F) staining, and immunohistochemistry (IHC). Scoring was performed using a histological grading method (see Table S5 for specific scoring criteria) based on available studies. The relative positivity of IHC was analysed by ImageJ.^57^

### X-ray and MRI examination

At 4 and 8 weeks post-surgery, six randomly selected rats from each group were anaesthetised with 10% sodium pentobarbital (40 mg/kg) through intraperitoneal injection. The rats were then positioned prone for X-ray and MRI scans. X-ray exposure parameters were set to 50 Kv and 160 μA. MRI images were obtained using a 3.0 T MRI system (Philips Intera Achieva) to assess disc signal and structural alterations. Three independent orthopaedic investigators evaluated the extent of disc degeneration using both MRI and X-ray images, employing the Pfirrmann (See Table S6 for specific criteria) and DHI grading systems.^57^

### Statistical analysis

Data are shown as mean ± SD from three or more experiments. Statistical analysis was conducted with Graphpad Prism 8.0.1 (Graphpad Inc., La Jolla, CA, USA), using two-tailed Student’s *t*-tests and two-way ANOVA with Tukey–Kramer post-tests to assess significance. *P*-values < 0.05 were significant, while those >0.05 were not (*P < 0.05, **P < 0.01, ***P < 0.001, ****P < 0.000 1). Results were validated across three independent experiments.

More supplementary Materials and Methods are available in Supplementary Information.

## Supplementary information

Supporting Information

## Data Availability

All data are available upon reasonable request from the corresponding author with the publication.
