# Supplementary material for: KMT2A regulates the autophagy-GATA4 axis through METTL3-mediated m6A modification of ATG4a to promote NPCs senescence and IVDD progression
Source: Bone Res. 2024 Nov 21;12:67. doi: 10.1038/s41413-024-00373-1 (PMC11582572; doi:10.1038/s41413-024-00373-1)
Supplement: Supplementary file 1 — Supporting Information [file 41413_2024_373_MOESM1_ESM.doc]

**Supplementary Information File**

**KMT2A regulates the autophagy-GATA4 axis through METTL3-mediated m^6^A modification of ATG4a to promote NPCs senescence and IVDD progression.**

Ouqiang Wu^#,1^, Yuxin Jin^#,1^, Zhiguang Zhang^#,1^, Hao Zhou^1^, Wenbin Xu^2^, Linjie Chen^1^, Morgan Jones^4^, Kenny Yat Hong Kwan^5^, Jianyuan Gao^1^, Kai Zhang^6^, Xiaofei Cheng^6^, Qizhu Chen^1^, Xinzhou Wang^1^, Yan Michael Li^7^, Zhenyu Guo^1^, Jing Sun^1^, Zhihua Chen^1^, Bin Wang^3^, Xiangyang Wang^1^, Shuying Shen^2*^, Aimin Wu^1*^

#These authors contributed equally: Ouqiang Wu, Yuxin Jin, Zhiguang Zhang.

*Corresponding Authors: Shuying Shen; Aimin Wu.

E-mail: 11207057@zju.edu.cn (Shuying Shen); aiminwu@wmu.edu.cn (Aimin Wu)

**This file includes:**

Supplementary Materials and Methods

Supplementary Tables 1 to 6

Supplementary Figures 1 to 14

**Supplementary Materials and Methods**

**Ethics Approval and Consent to Participate**

All protocols involving patient samples received approval from Wenzhou Medical University's Ethics Committee (No. 2022057). Similarly, all animal model protocols were approved by the Experimental Animal Ethics Committee of Wenzhou Research Institute of State Science and Technology (No. WIUCAS23020208). The design of the entire study conformed to the principles outlined in the Declaration of Helsinki.

**Clinical samples**

The human NP tissue employed in this study was sourced from patients at the Second Affiliated Hospital of Wenzhou Medical University and received approval from the ethics committee (No. 2022057). All patients provided their signed informed consent. The degree of IVDD was assessed by MRI and graded according to the Pfirrmann grading system. The degenerative intervertebral disc group was further subdivided into groups II, III, IV, and V. The study cohort comprised patients aged 45 to 65 years (n = 20; average age 47.3 years).

**Western blot**

NPCs were lysed on ice with RIPA buffer and 1 mM PMSF (Beyotime, China), then centrifuged at 12,000 rpm for 30 minutes at 4 °C to collect total proteins. Protein concentrations were measured using Beyotime's Micro-BCA Protein Assay Kit. Proteins were separated by electrophoresis on 8%-12% SDS-polyacrylamide gels (Vazyme Biotech, China) and transferred onto PVDF membranes (Bio-Rad, the United States of America). Membranes were blocked with Beyotime's rapid solution for 15 minutes at room temperature, incubated with primary antibodies overnight at 4 °C (**Table S1** for antibody details), and then with HRP-conjugated goat anti-rabbit or anti-mouse IgG (Proteintech, China). GAPDH or H3 were loading controls. Bands were detected using Affinity's ECL reagent and analyzed with ImageJ.

**Cell culture**

NP tissues derived from patients during surgery were initially dissected into small pieces and then digested with a 0.2% type II collagenase solution (Invitrogen, the United States of America) at 37°C for 8 hours. Subsequently, the cells from the digested material were gathered by centrifuging at 1200 rpm for 5 minutes and cultured in DMEM/F12 medium (Gibco, the United States of America) supplemented with 100 μg/mL streptomycin, 100 U/mL penicillin, and 10% fetal bovine serum (FBS) (Invitrogen, the United States of America). The cell cultures were maintained at 37°C with 5% CO2, with medium changes every two days.

**qRT-PCR for RNA Expression Level.**

NPCs total RNA was isolated using Beyotime's Total RNA Extraction Kit (China) and converted to cDNA with Takara's PrimeScript RT kit (Japan). The cDNA underwent qRT-PCR with Takara's TB green kit (Japan) on a Roche LightCycler® 96 (Germany), normalizing to GAPDH as an internal control. Expression levels were determined via the 2-ΔΔCt method.The primer sequences used are listed in **Table S2.**

**SA-β-gal staining**

Cellular senescence levels were evaluated using the SA-β-gal staining kit (Beyotime, China) according to the manufacturer's instructions. Senescent cells demonstrated increased SA-β-gal enzymatic activity, which resulted in a distinctive blue stain. To quantify the senescent cells, twenty-five random fields of view were selected from each slide, and the number of SA-β-gal-positive cells was counted using a Nikon ECLIPSE Ti microscope.

**EdU incorperation assay**

NPCs proliferation was evaluated using Beyotime's BeyoClick™ EdU Staining Kit (China) following the included guidelines. NPCs in a 6-well plate were treated with 20 μM EdU and incubated at 37°C for 2 hours. Post-incubation, cells were fixed in 4% paraformaldehyde for 15 minutes, permeabilized with 0.3% Triton X-100 in PBS for 10 minutes and washed with PBS thrice. They were then incubated with Click solution (430 μl Click Reaction Buffer, 20 μl CuSO4, 1 μl Azide 594, 50 μl Click Additive Solution) for 30 minutes in darkness. DNA was stained with Hoechst 33342 for 30 minutes. An Olympus BX53 microscope captured the images, and ImageJ software quantified EdU-positive cells. The procedure was performed in triplicate for reliability.

**Behavioural testing**

The Hargreaves test places the rat alone in an opaque experimental box for half an hour to eliminate environmental disturbances. After acclimatisation, the rats were irradiated with a radiant light source at the tail puncture point and the latency of thermal contraction foot was calculated to assess the thermal nociceptive threshold. The experiments were performed on postoperative days 7 and 14, and each rat was measured six times at each time point, and the mean reaction time was calculated as the final value.The Von Frey test was performed by placing the rats individually in an opaque experimental box for half an hour to eliminate environmental disturbances. After acclimatisation, the tail of the mouse was pressed with a Von Frey filament, and a positive response was defined when the mouse showed behaviours such as licking, flinching and tail wagging in response to the stimulus. Measurements were taken 6 times each with different weights of filaments, and the weight of the filaments at the time of 50% positive response was recorded as the mechanical pain threshold. This experiment was conducted on the 7th and 14th postoperative days, respectively.

**m^6^A Level Quantification Assay**

Elisa-based m6A colourimetric analysis was performed using the EpiQuik m6A RNA Methylation Quantification Kit (colorimetric) (Epigentek) according to the manufacturer's protocol for quantification of m6A levels in measured mRNA.

**RNA pull-down.**

Biotinylated DNA probes containing T7 and SP6 promoters complementary to ATG4a were synthesised and dissolved in 500 μl lysis buffer (0.5 M NaCl, 20 mM Tris-HCl, pH 7.5 and 1 mM EDTA). The following RNA Pulldown assay steps were then performed according to the instructions of the manufacturer of the MagCapture™ RNA Pull Down kit (Millipore Corporation, USA). Cell lysates were incubated with probe-coated beads. The pulldown mixture was then used for subsequent protein blot analysis.

**RNA Stability Assay**

After being treated with 5 μg/ml actinomycin D (MedChem Express) for the inhibition of mRNA transcription, cells were collected at 0, 2, 4, 6, and 8 hours to analyze mRNA levels and the rate of degradation. Total RNA was extracted and employed for RT-qPCR. The degradation rate of RNA (k) was computed using the equation: e^-kt^ = N0/Nt, where t indicates the time after transcription inhibition and k represents the degradation rate, and Nt and N0 are the relative mRNA expression at time t and time 0, respectively. The RNA half-life (t1/2) was calculated from the degradation rate as t1/2 = ln2/k.

**RNA Interference and Plasmid Transfection**

Knockdown of GATA4, ATG4a, METTL3, YTHDF1, YTHDF2 and KMT2A in NPCs was realized by transfection with siRNA. siRNA against GATA4 (siGATA4), ATG4a (siATG4a), METTL3(siMETTL3), YTHDF1(siYTHDF1), YTHDF2 (siYTHDF2) and KMT2A (siKMT2A) and scrambled siRNA (siControl) were synthesized by [GenePharma](https://www.bing.com/ck/a?!&&p=5c3356ce06a211bfJmltdHM9MTcyMTE3NDQwMCZpZ3VpZD0wNTllYjlmZC00NzE1LTZmMjktMjM1Ny1hYWNhNDY3MzZlYzcmaW5zaWQ9NTIwNw&ptn=3&ver=2&hsh=3&fclid=059eb9fd-4715-6f29-2357-aaca46736ec7&psq=吉玛生物&u=a1aHR0cDovL3d3dy5nZW5lcGhhcm1hLmNvbS8&ntb=1) (Shanghai, China) and transfected with Lipofectamine 2000 (Invitrogen) according to the standard protocol. The siRNA sequences were listed in **Table S4**. After verified high silencing efficiency, the NP cells were then used in following treatment. Vectors of adeno-associated virus pLKO.1 were used in which shRNA against GATA4(*Mouse*) were cloned. And the targeted sequences of shRNA were listed in the Supplementary Material **Table S4**. pGL3-GATA4, pGL3-ATG4a, pGL3-METTL3, pGL3-YTHDF2 and pGL3-KMT2A plasmids were constructed followed by lentivirus package by GenePharma (Shanghai, China) for overexpression experiments.

**Supplementary Tables**


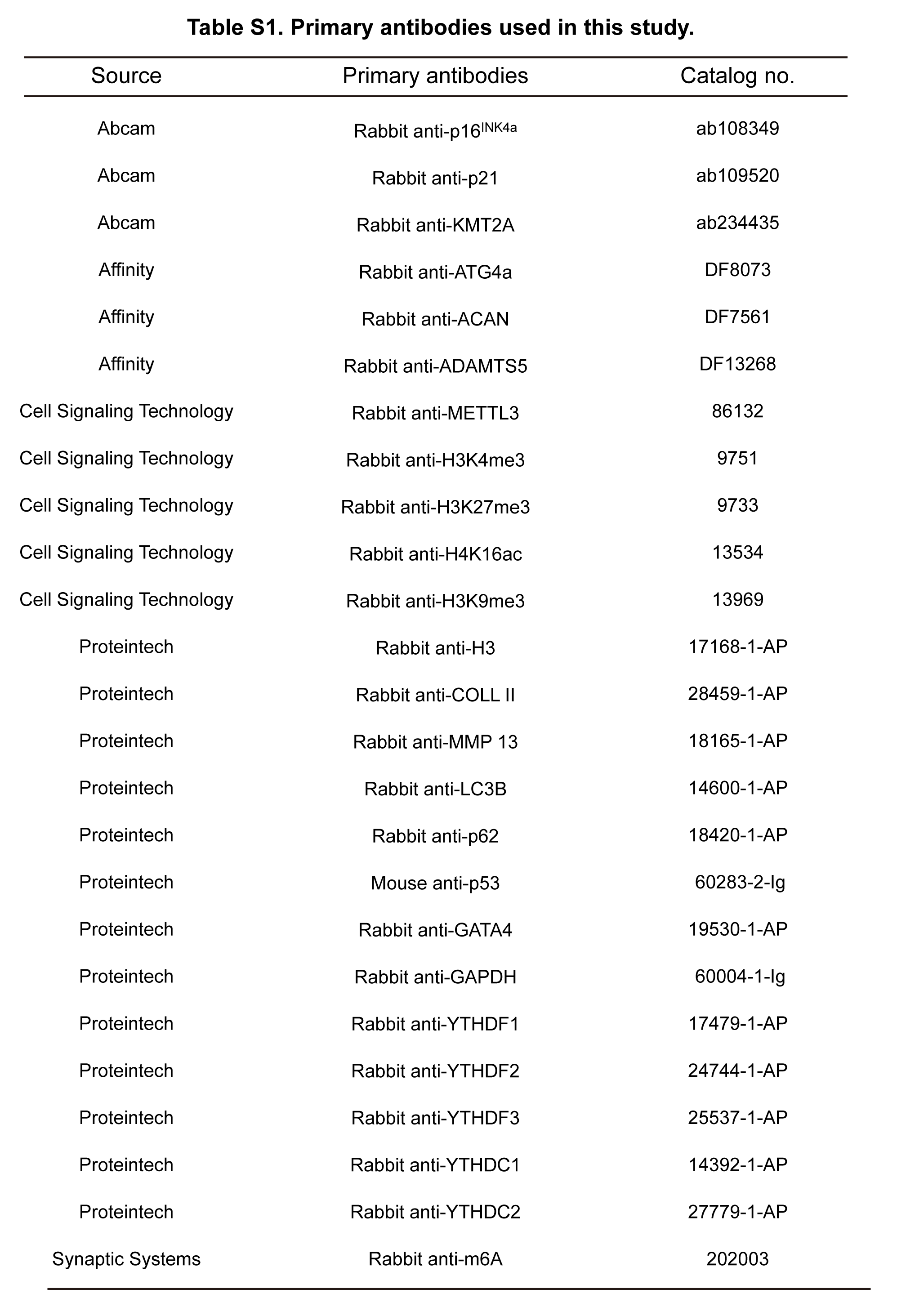

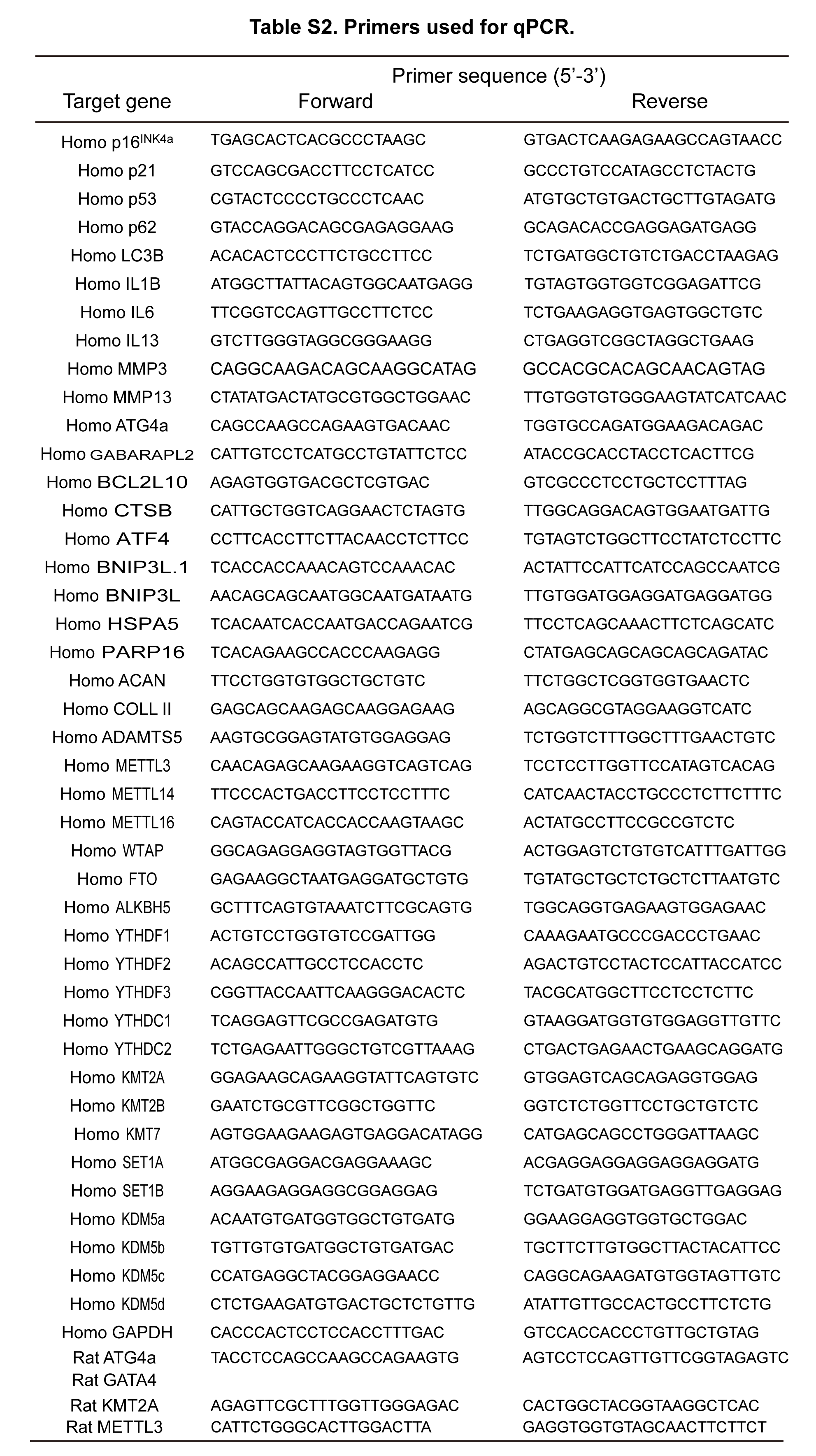

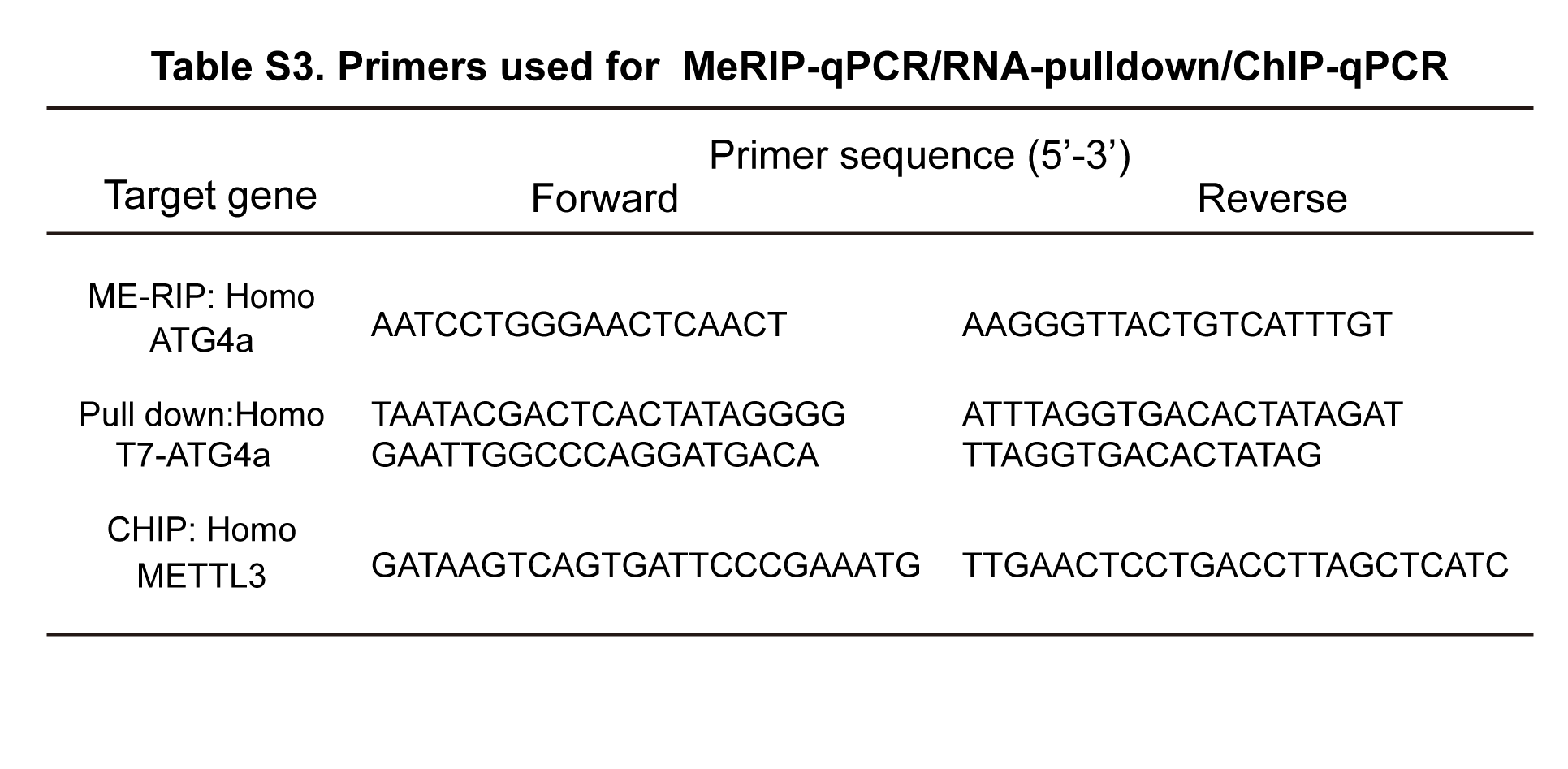


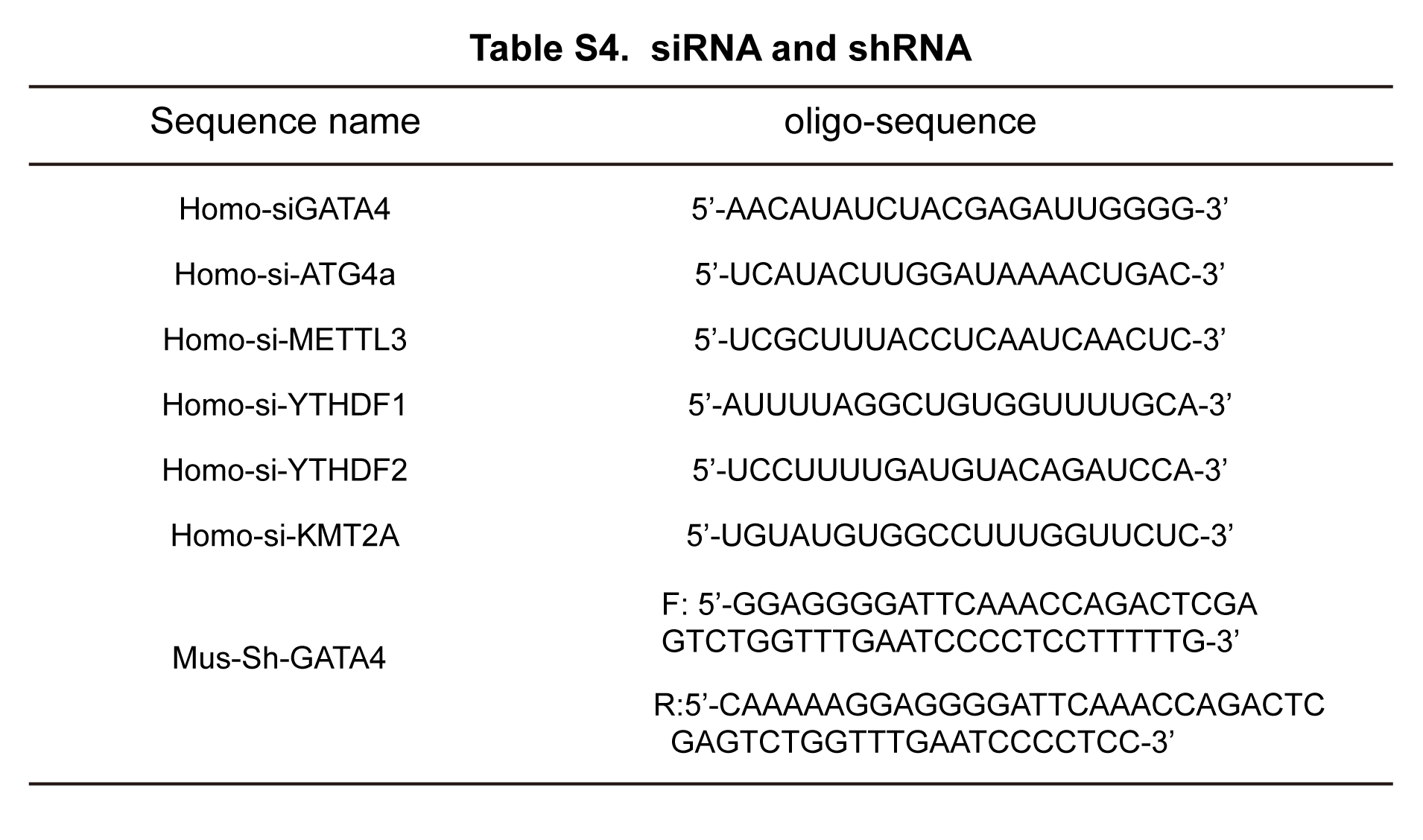

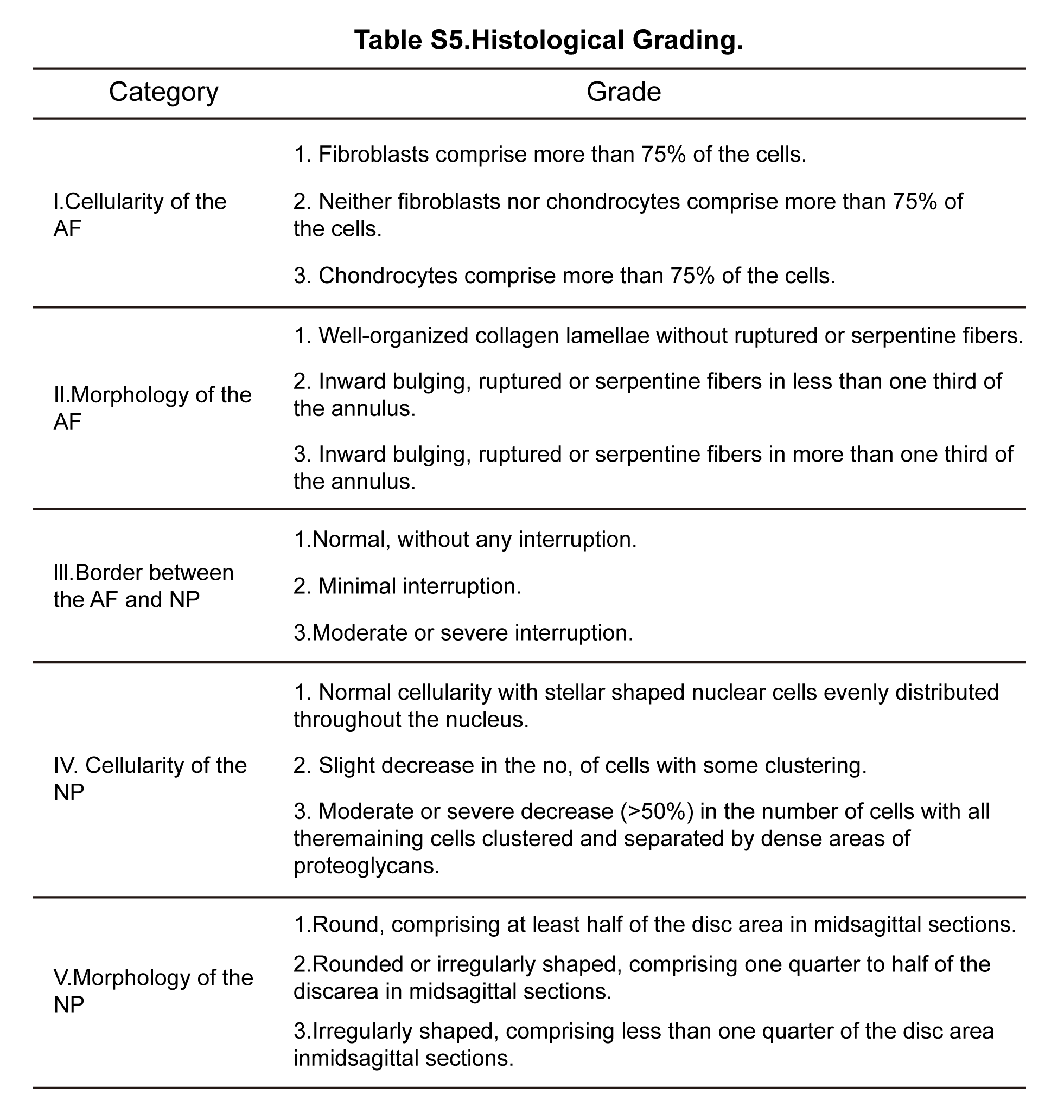

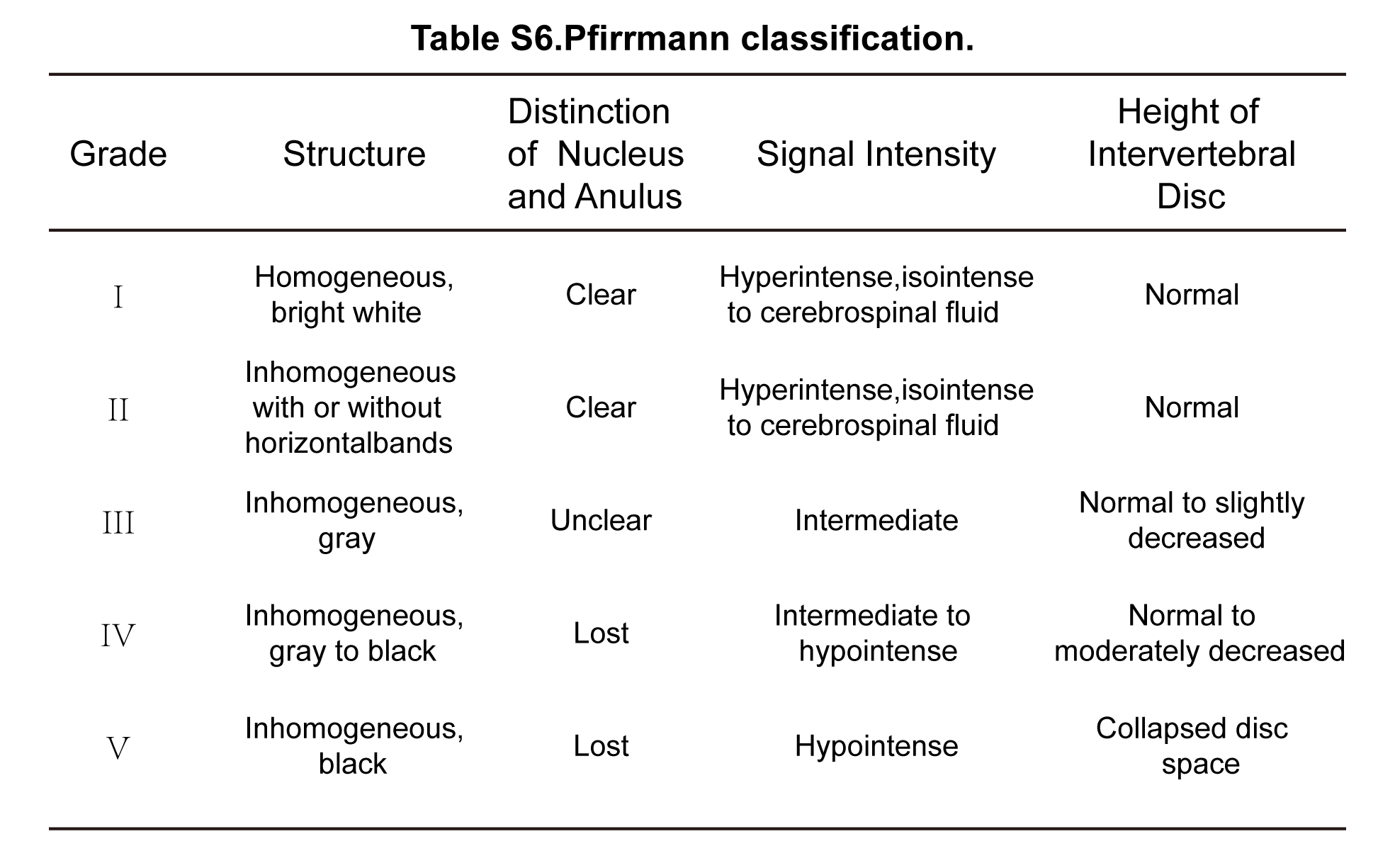


**Supplementary Figures**


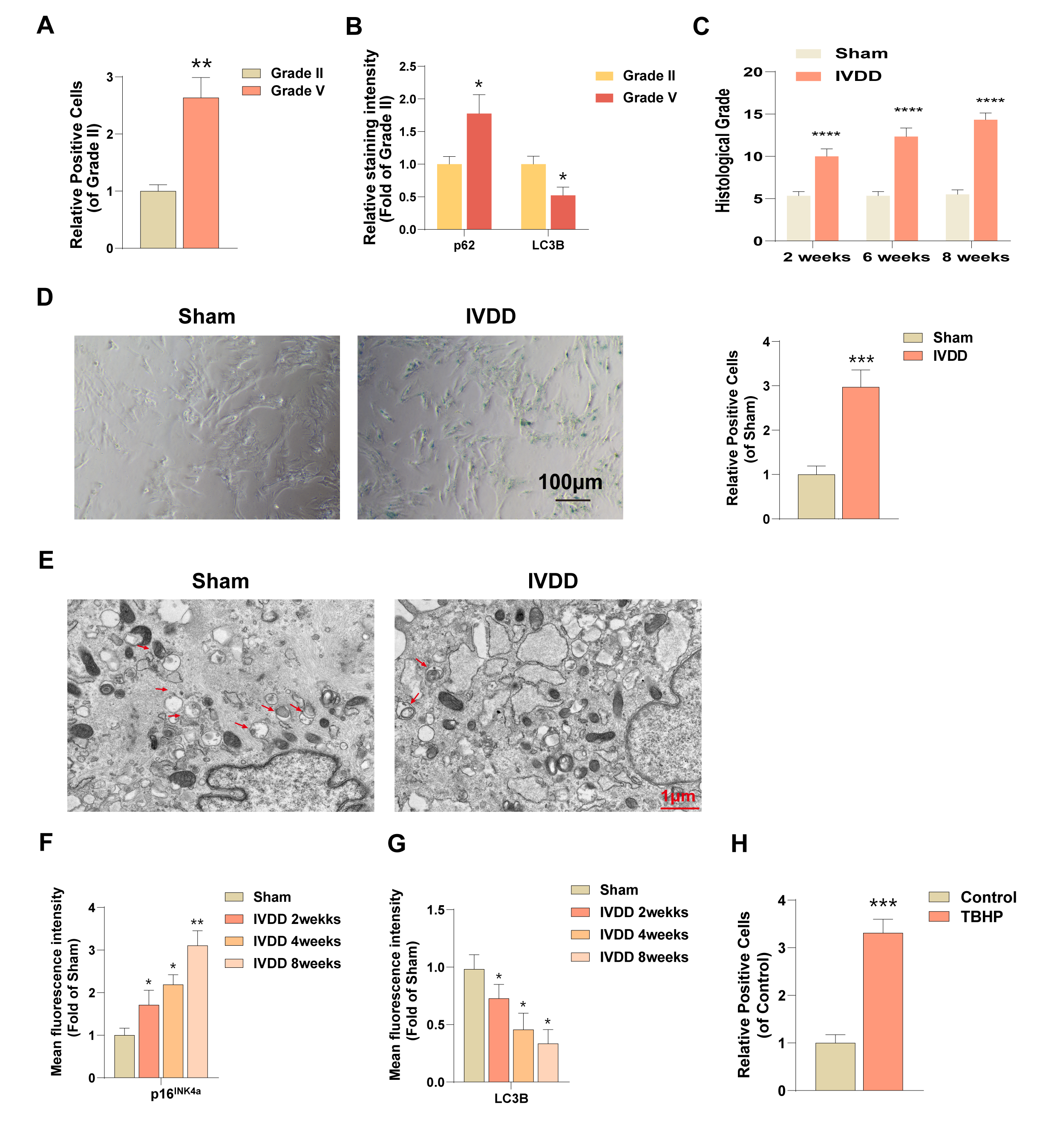


**Figure S1.(A)** Quantitative Analysis of SA-β-gal Staining in Grade II and Grade V NPCs( N = 3).**P < 0.01.**(B)** Quantitative Analysis of Immunohistochemical Staining in Grade II and Grade V NP Tissues(N = 6).*P < 0.05.**(C)** Histological sections were graded using the Histological Grades System and analyzed for the severity of the IVDD-like phenotype (N = 6).****P < 0.0001.**(D)** Number of positive cells in rat NPCs of Sham and IVDD origin detected by SA-β-gal (N = 3).***P < 0.001.**(E)** Representative transmission electron microscopy images were acquired of rat normal and degenerated NPCs. Red arrows highlight autophagic vesicles. **(F,G)** Quantitative analysis of p16^INK4a^ and LC3B protein immunofluorescence staining in NP tissues(N = 6).*P < 0.05,**P < 0.01.**(H)** Quantitative Analysis of SA-β-gal Staining in normal and TBHP-stimulated NPCs (N = 3). ***P < 0. 001.Data are expressed as mean ± SD.


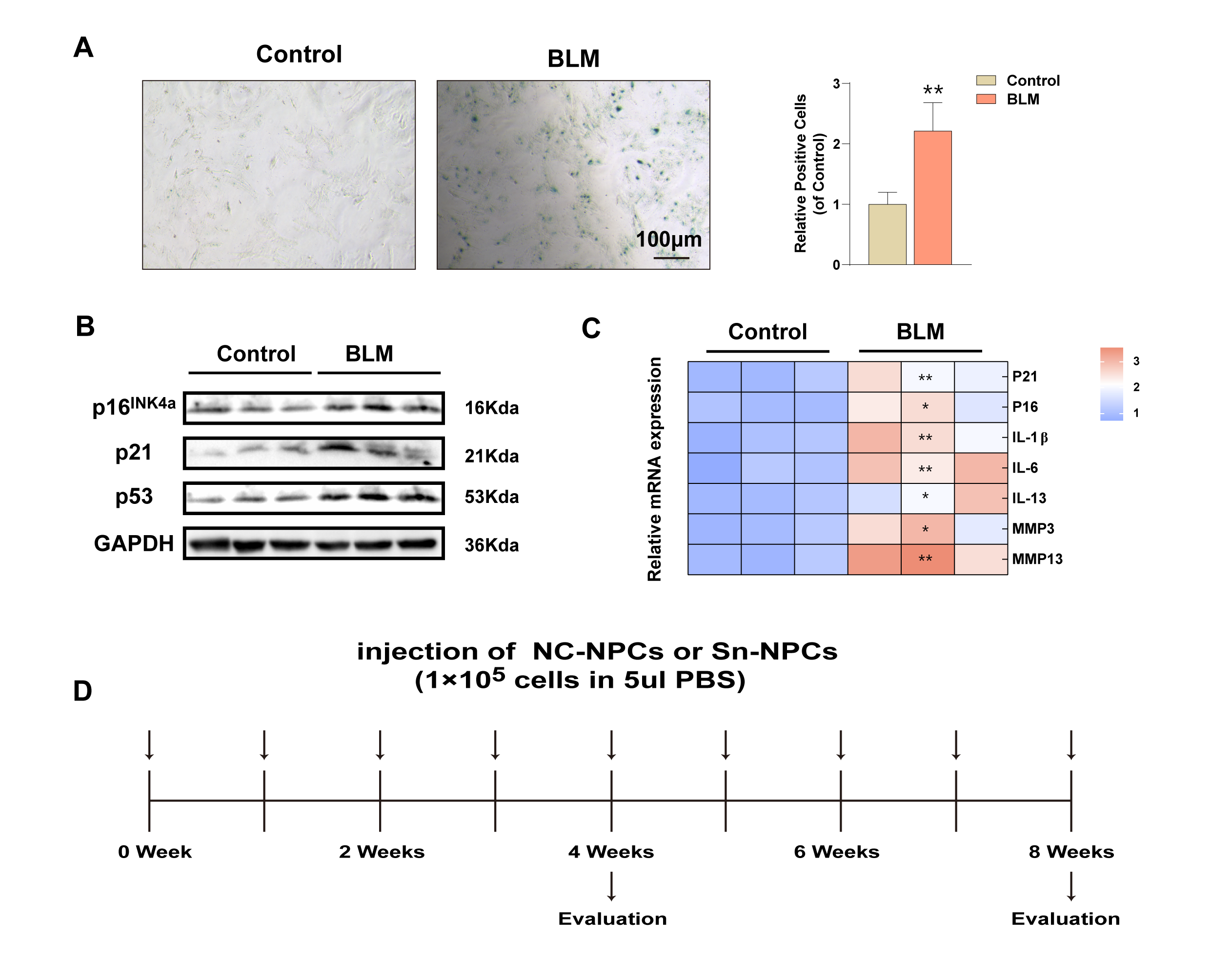


**Figure S2. (A)** The SA-β-Gal staining in normal and bleomycin treatment NPCs(N = 3).**P < 0.01. **(B)** Westernblot analysis of pl6^INk4a^ and p21 protein levels in NPCs with or without BLM. **(C)** qPCR analysis of mRNA levels of SASP-associated inflammatory factors (IL-1β, IL-6, IL-13), matrix metalloproteinases (MMP3 and MMP13) and cellular senescence markers (p16^INK4a^ and p21) in the indicated groups(N = 3).*P < 0.05,**P < 0.01. **(D)** Experimental design diagram for the injection of normal NPCs (NC-NPCs) or bleomycin-induced senescent NPCs (Sn-NPCs) into rat intervertebral discs. Data are expressed as mean ± SD.


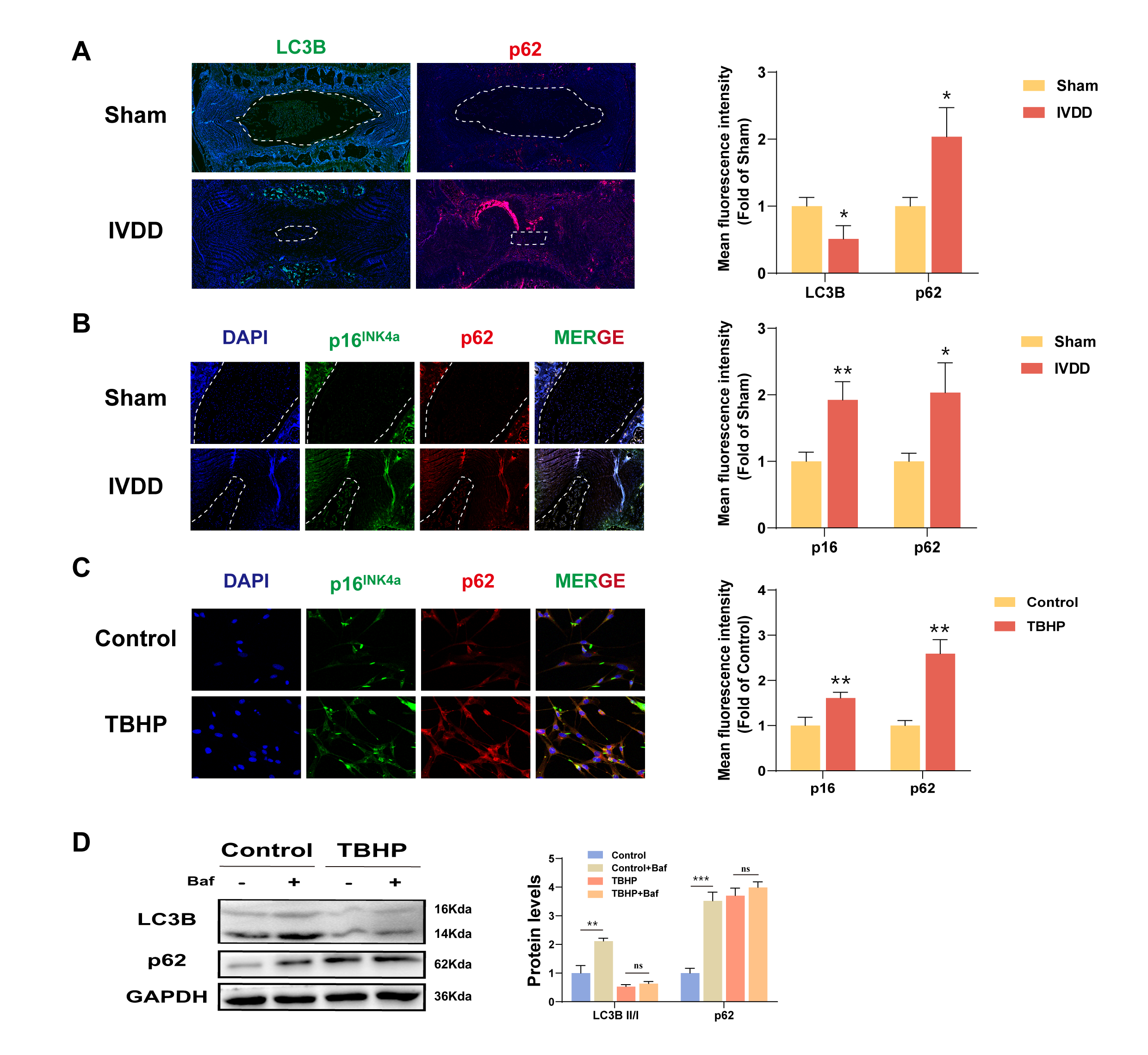


**Figure S3. (A)** Representative plots of double fluorescence immunostaining of LC3B and p62 proteins in NP tissues of rats and sham-operated rats 8 weeks after IVDD surgery (N = 6). *P < 0.05. **(B)** Representative plots of double fluorescence immunostaining for p16^INK4a^ and p62 proteins in NP tissues from rats and sham-operated rats 8 weeks after IVDD surgery (N = 6). *P < 0.05,**P < 0.01. **(C)** Representative images of double fluorescence immunostaining for p16^INK4a^ and p62 proteins in normal and senescent NPCs induced by TBHP (N = 3). **P < 0.01.**(D)** Western blot analysis of LC3B and p62 protein levels in normal or senescent NPCs treated with bafilomycin A1 (Baf, 50 nM) for 48 hours (N = 3). Data are expressed as mean ± SD. ns > 0.05, **P < 0.01, ***P < 0.001.


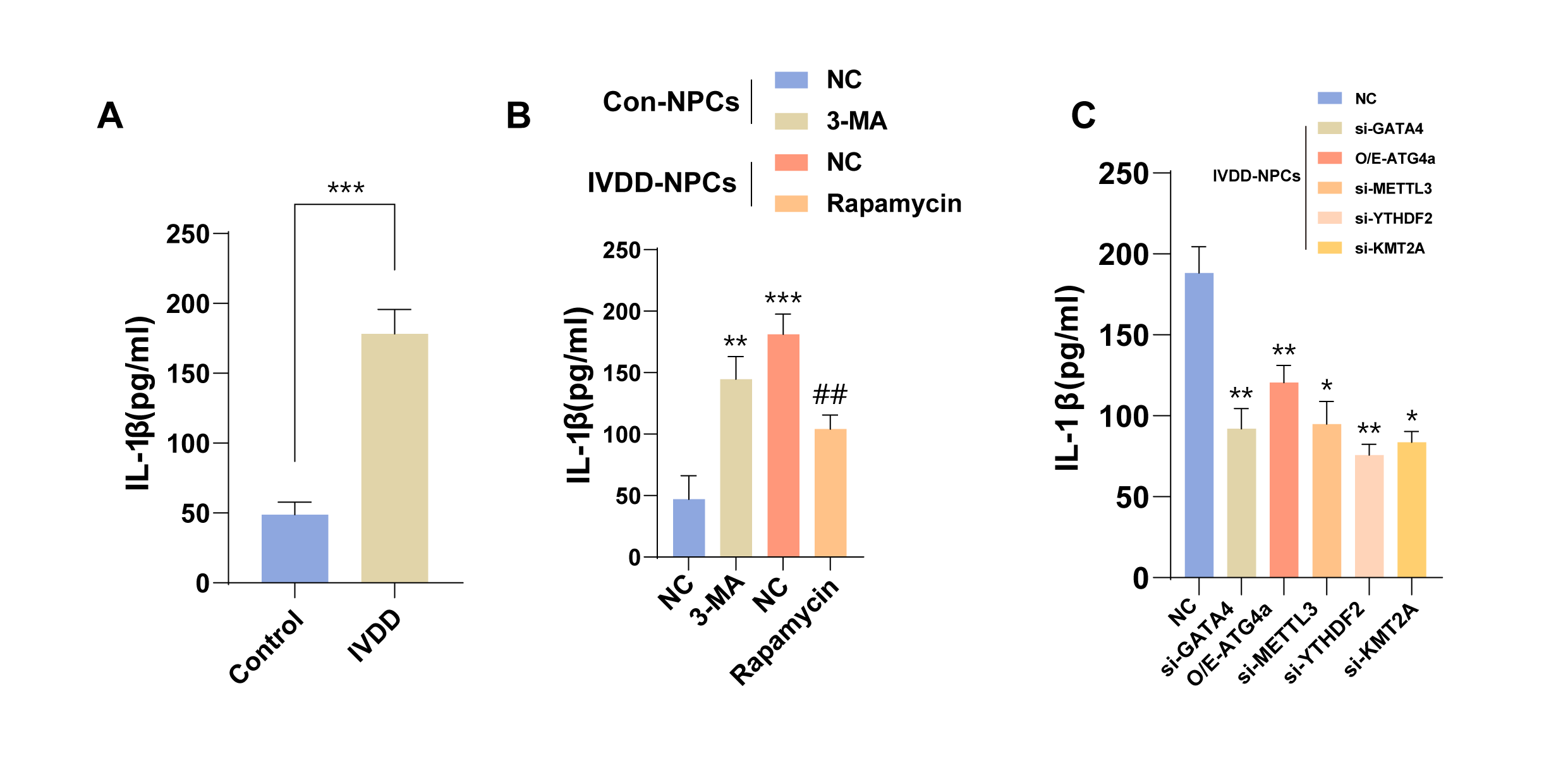


**Figure S4. (A)** IL-1β levels in supernatants of Con-NPCs or IVDD-NPCs (N = 3). ***P < 0.001. **(B)** IL-1β levels in supernatants of Con-NPCs transfected with 3-MA (1 mM) and IVDD-NPCs treated with rapamycin (50 nM) (N =3). **P < 0.01, ***P < 0.001, * VS Con-NPCs+NC; ##P < 0.01, # VS IVDD-NPCs+NC. **(C)** IVDD-NPCs were transfected with siRNAs targeting GATA4 (si-GATA4), targeting pcDNA3.1-ATG4avector (O/E-ATG4a) (N = 3), IL-1β levels in supernatants of siRNA targeting METTL3 (si-METTL3), or siRNA targeting KMT2A (si-KMT2A). Data are expressed as mean ± SD.


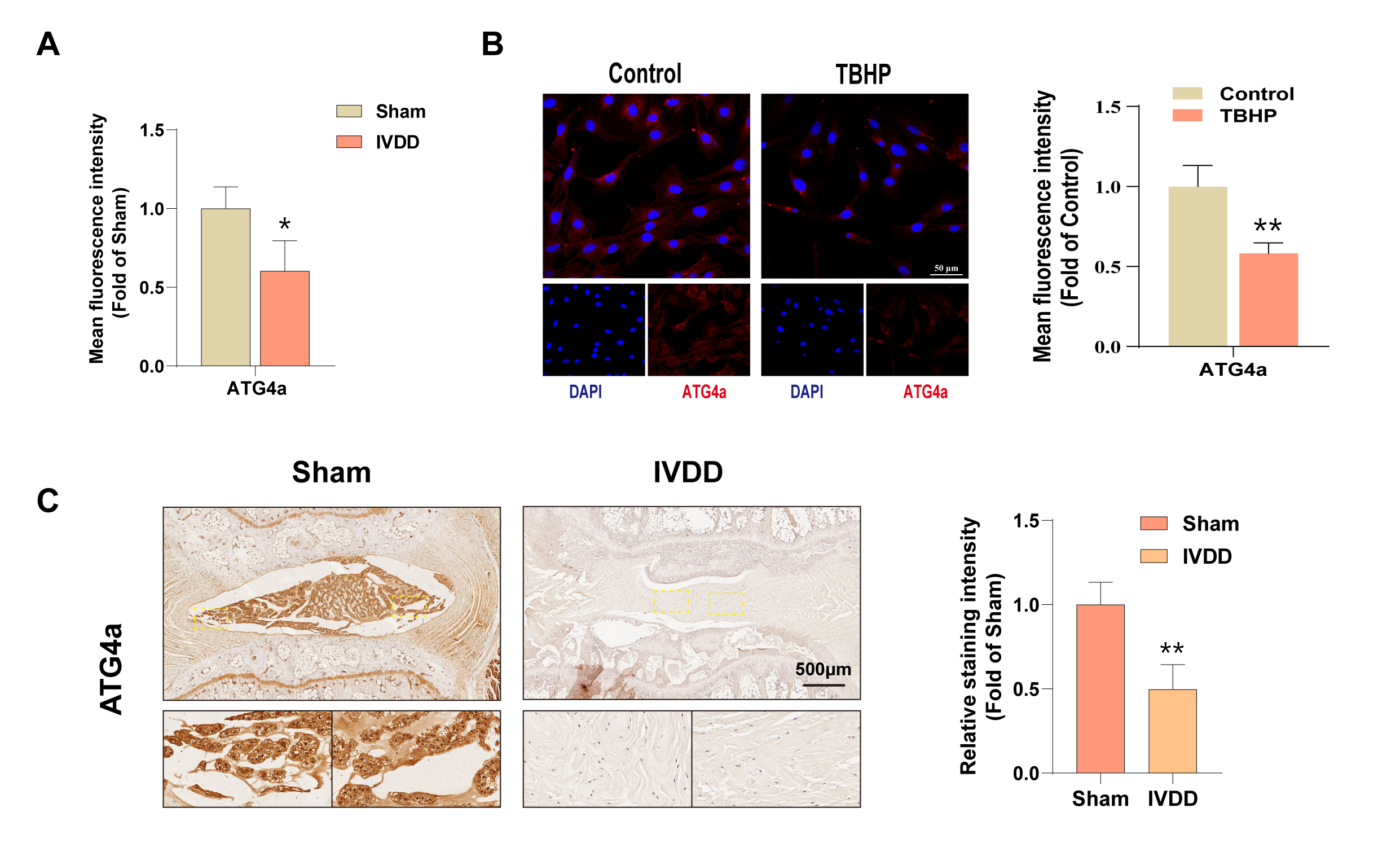


**Figure S5.(A)** Quantitative analysis of ATG4a protein immunofluorescence staining in NP tissues(N = 6). *P < 0.05. **(B)** Representative images of fluorescence immunostaining of ATG4a protein in normal and senescent NPCs induced by TBHP (N = 3). **P < 0.01. **(C)** IHC analysis determined the protein levels of ATG4a in NP tissues (N = 6). **P < 0.01. Data are expressed as mean ± SD.


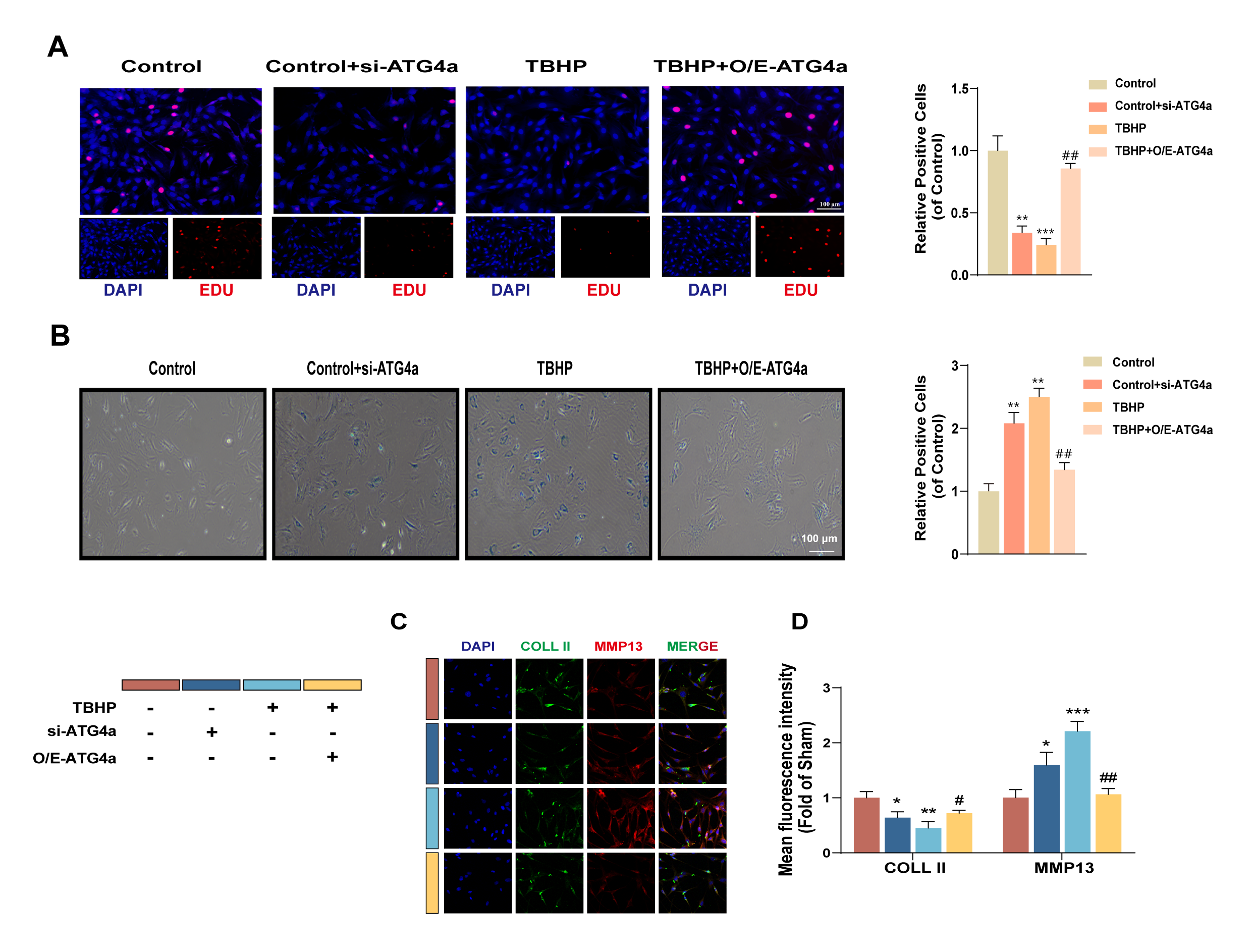


**Figure S6. (A)** Comparison of cell proliferation ability after silencing or overexpressing ATG4a in normal NPCs and senescent NPCs induced by TBHP using EdU method (N = 3). **P < 0.01, **P < 0.001, ***P < 0.001, * VS Control; ##P < 0.01, # VS TBHP. **(B)** Comparison of the number of senescent cells after silencing or overexpression of ATG4a in normal NPCs and senescent NPCs induced by TBHP (N = 3) using SA-β-gal staining. **P < 0.01, ***P < 0.001, * VS Control; ##P < 0.01, # VS TBHP. **(C)** Representative images of double fluorescence immunostaining for COLL II and MMP 13 proteins in NPCs (N = 3). *p < 0.05, **P < 0.01, **P < 0.001, ***P < 0.001, * VS Control; #p<0.05, ##P < 0.01, # VS TBHP. Data are expressed as mean ± SD.


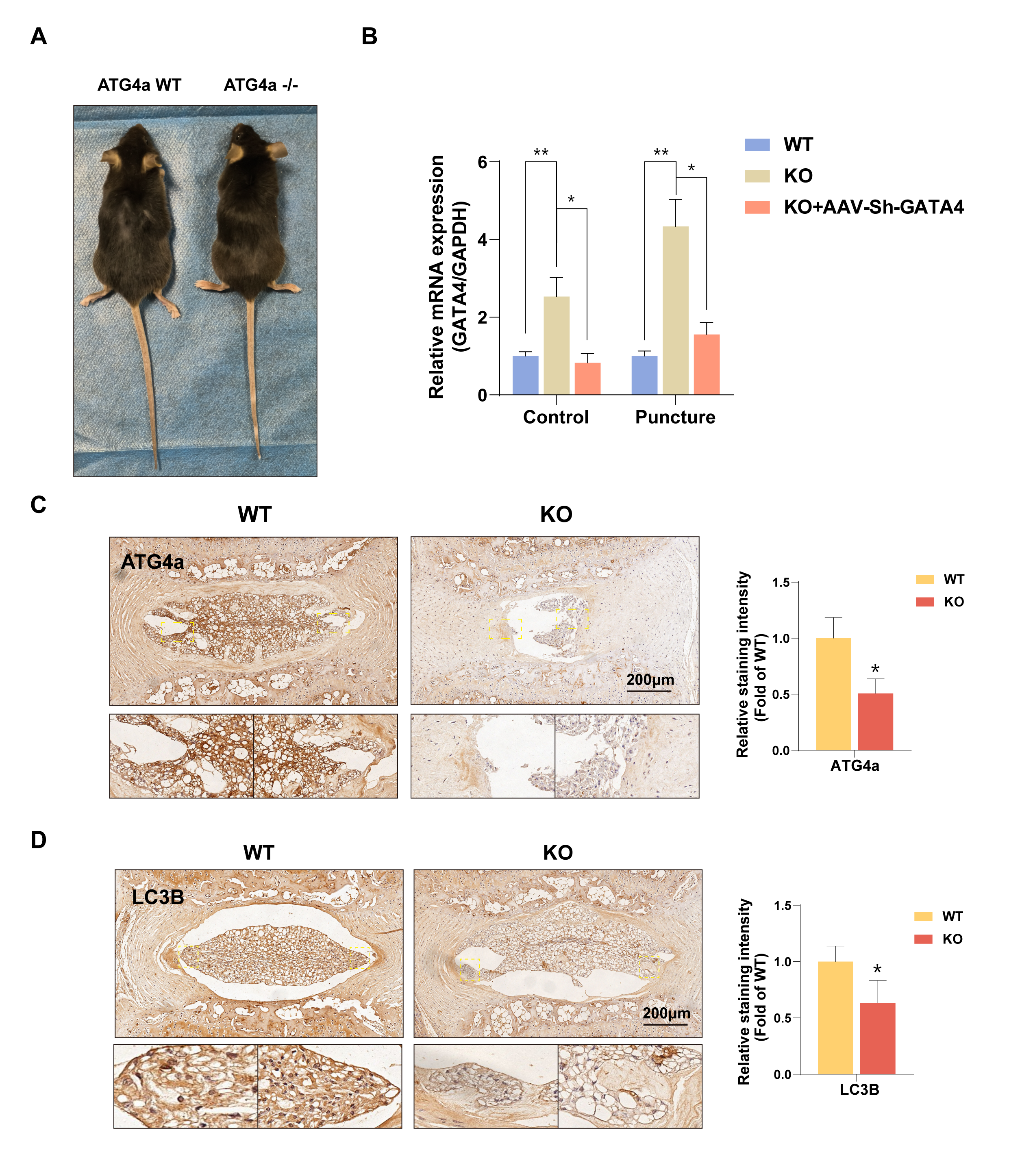


**Figure S7. (A)** ATG4a WT and ATG4a /- mice. **(B)** qPCR analysis of mRNA expression levels of ATG4a (N = 3). *P < 0.05,**P < 0.01. **(C)** The respective IHC staining of ATG4a in NP tissues of WT and KO mice (N = 6). *P < 0.05. **(D)** The respective IHC staining of LC3B in NP tissues of WT and KO mice (N = 6). *P < 0.05. Data are expressed as mean ± SD.


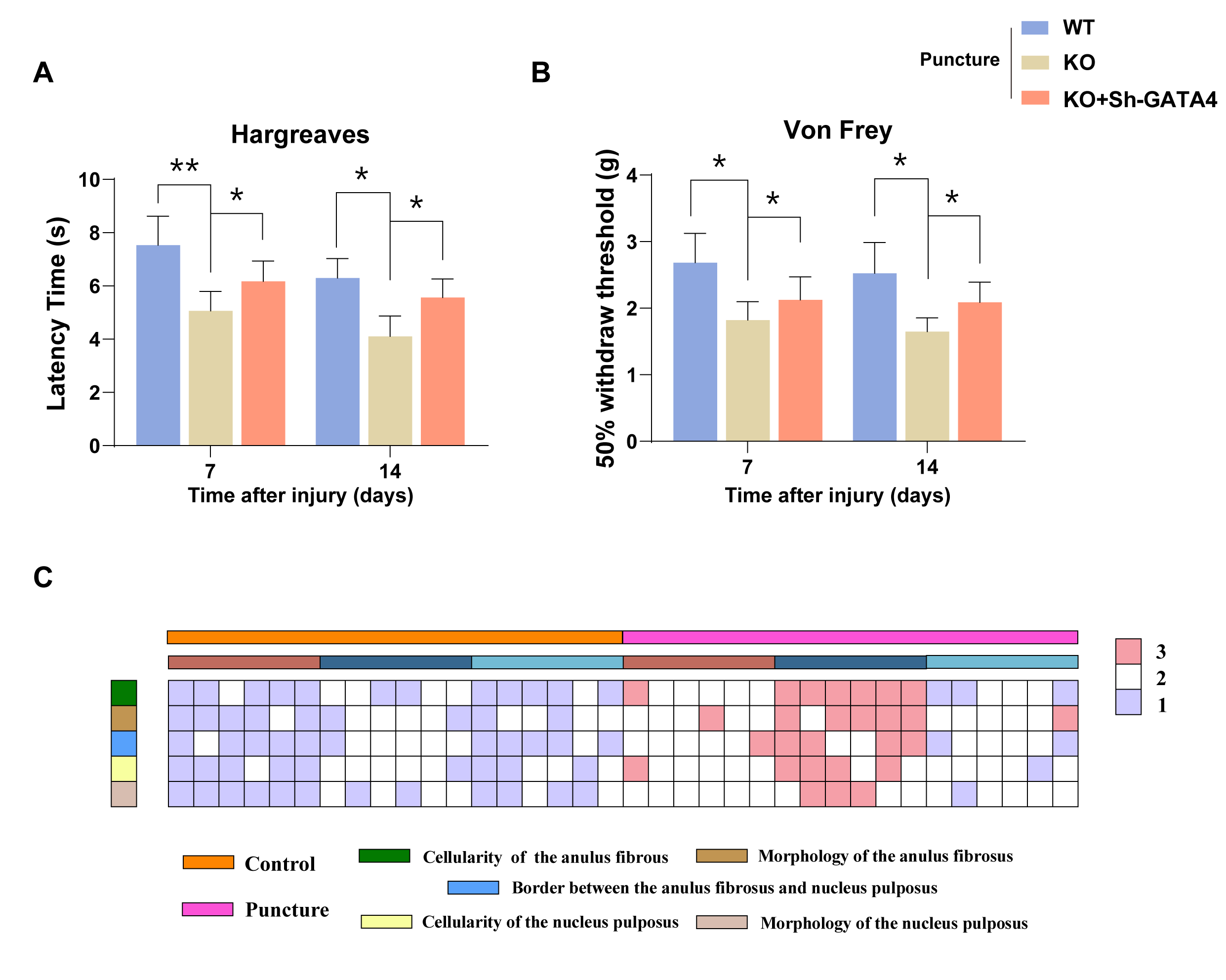


**Figure S8. (A)** Hargreaves test was performed to evaluate the thermal hyperalgesia of rats in various experimental groups(N = 6). *P < 0.05,**P < 0.01. **(B)** Von Frey test was performed to evaluate the mechanical allodynia of rats in various experimental groups(N = 6). *P < 0.05. **(C)** Histological scores of mose NP tissues. Data are expressed as mean ± SD.


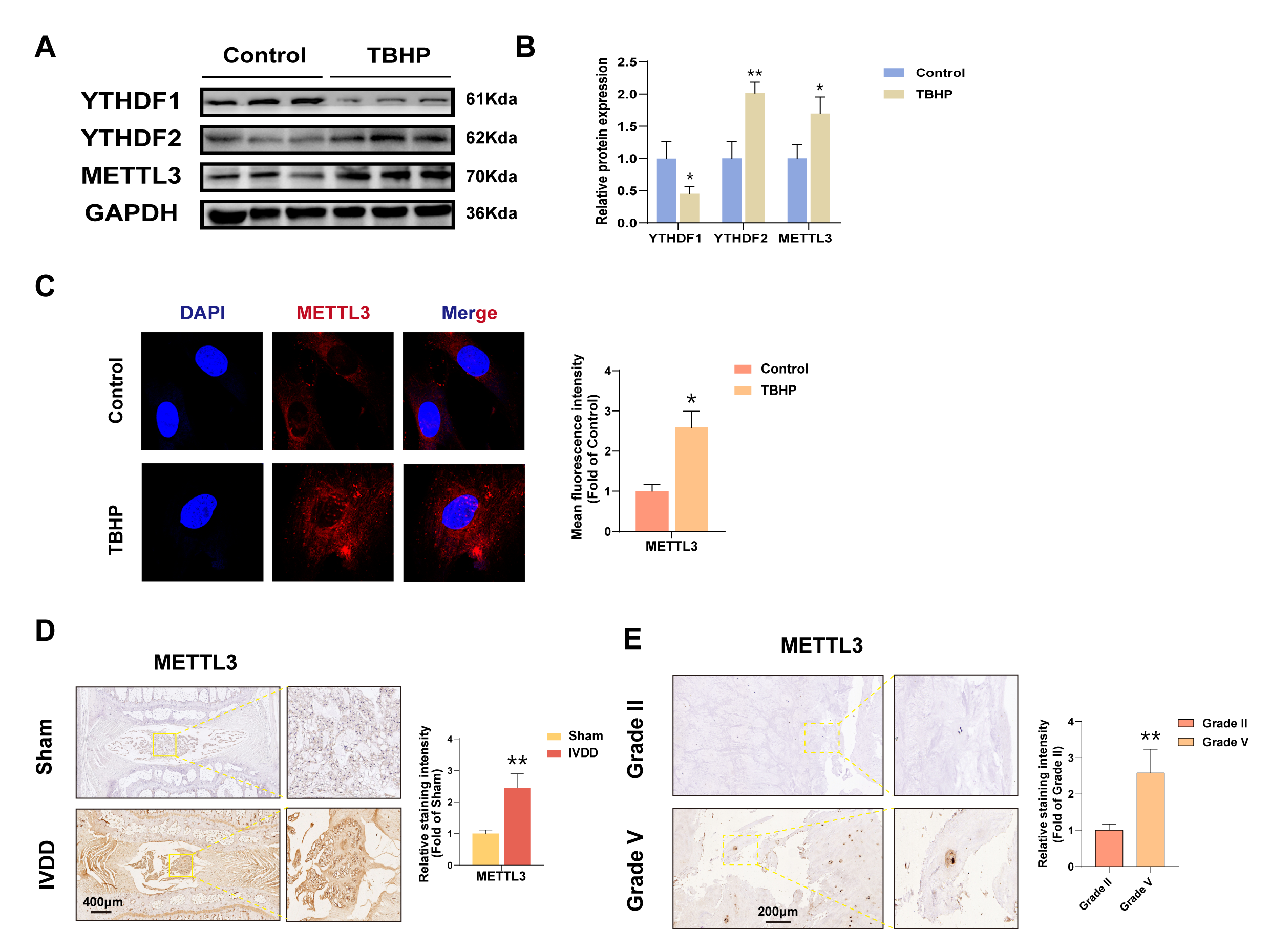


**Figure S9. (A, B)** Western blot analysis of protein expression levels of YTHDF1, YTHDF2 and METTL3 in normal NPCs and senescent NPCs induced by TBHP (N = 3). *P < 0.05,**P < 0.01. **(C)** IHC detection of METTL3 protein levels in NP tissues from rats 8 weeks after IVDD surgery and sham-operated rats (N = 3). *P < 0.05.**(D, E)** The respective IHC staining of METTL3 in NP tissues (N = 6). **P < 0.01. Data are expressed as mean ± SD.


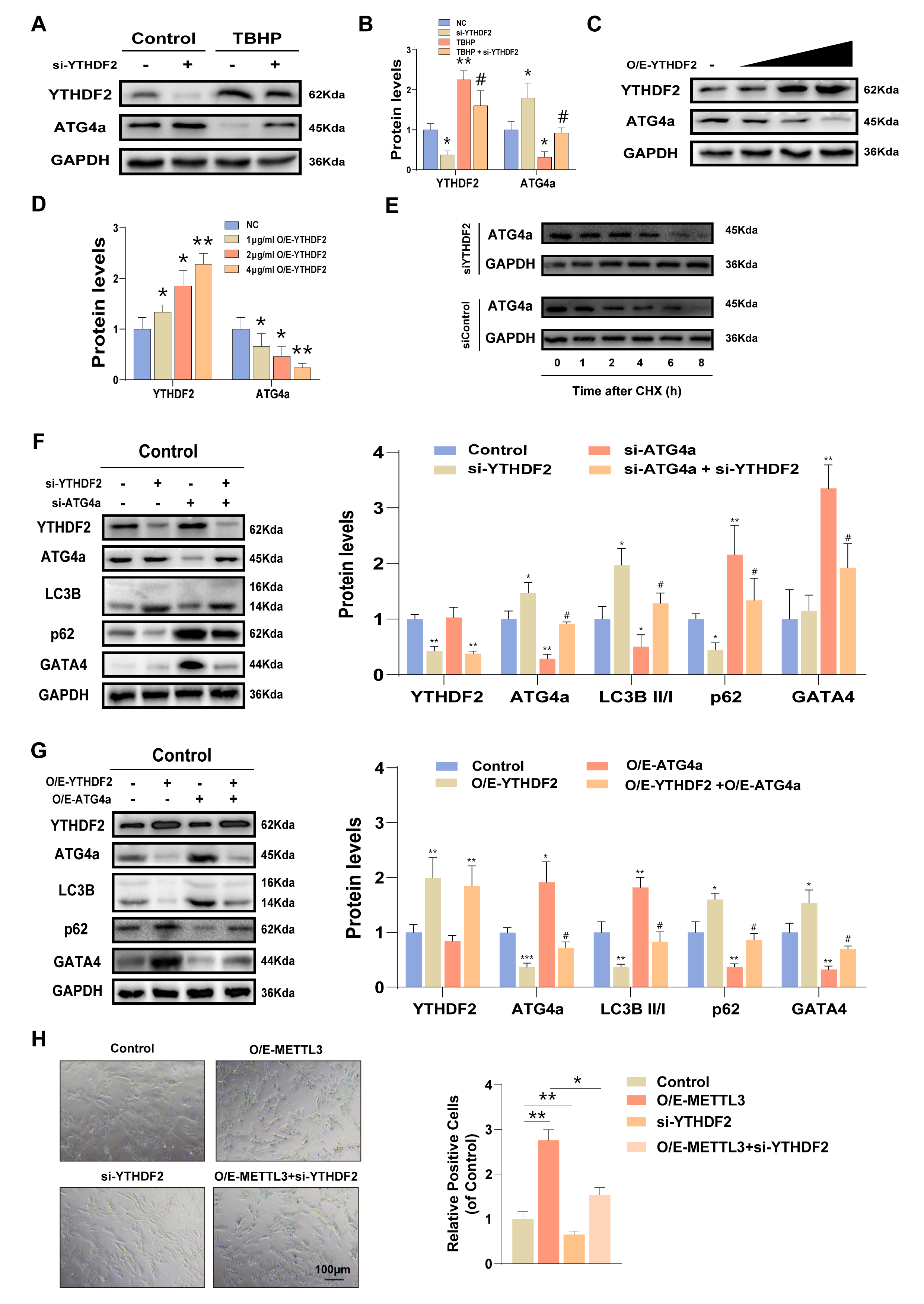


**Figure S10. (A, B)** Western blot analysis of YTHDF2 and ATG4a protein level after YTHDF2 knockdown (N = 3). *P < 0.05, **P < 0.01, * VS NC; #P < 0.05, # VS TBHP. **(C, D)** Western blot analysis of YTHDF2 and ATG4a protein level after overexpression of YTHDF2 (N = 3). *P < 0.05, **P < 0.01. **(E)** Protein stability analysis of ATG4a by western blot in NPCs with YTHDF2 silencing or not after CHX treatment. **(F)** Western blot analysis of protein levels of the indicated genes (YTHDF2, ATG4a, LC3B, p62 and GATA4) after silencing of YTHDF2 and ATG4a in Con-NPCs (N = 3). * P < 0.05, **P < 0.01, * VS Control; #P < 0.05, # VS si-YTHDF2. **(G)** Western blot analysis of protein levels of the indicated genes (YTHDF2, ATG4a, LC3B, p62 and GATA4) after overexpression of YTHDF2 and ATG4a in Con-NPCs (N = 3). *P < 0.05, **P < 0.01, * VS Control; #P < 0.05, # VS O/E-YTHDF2. **(H)** The representative images of SA-β-Gal staining for NPCs transfected with O/EMETTL3 or si-YTHDF2(N = 3). *P < 0.05, **P < 0.01. Data are expressed as mean ± SD.


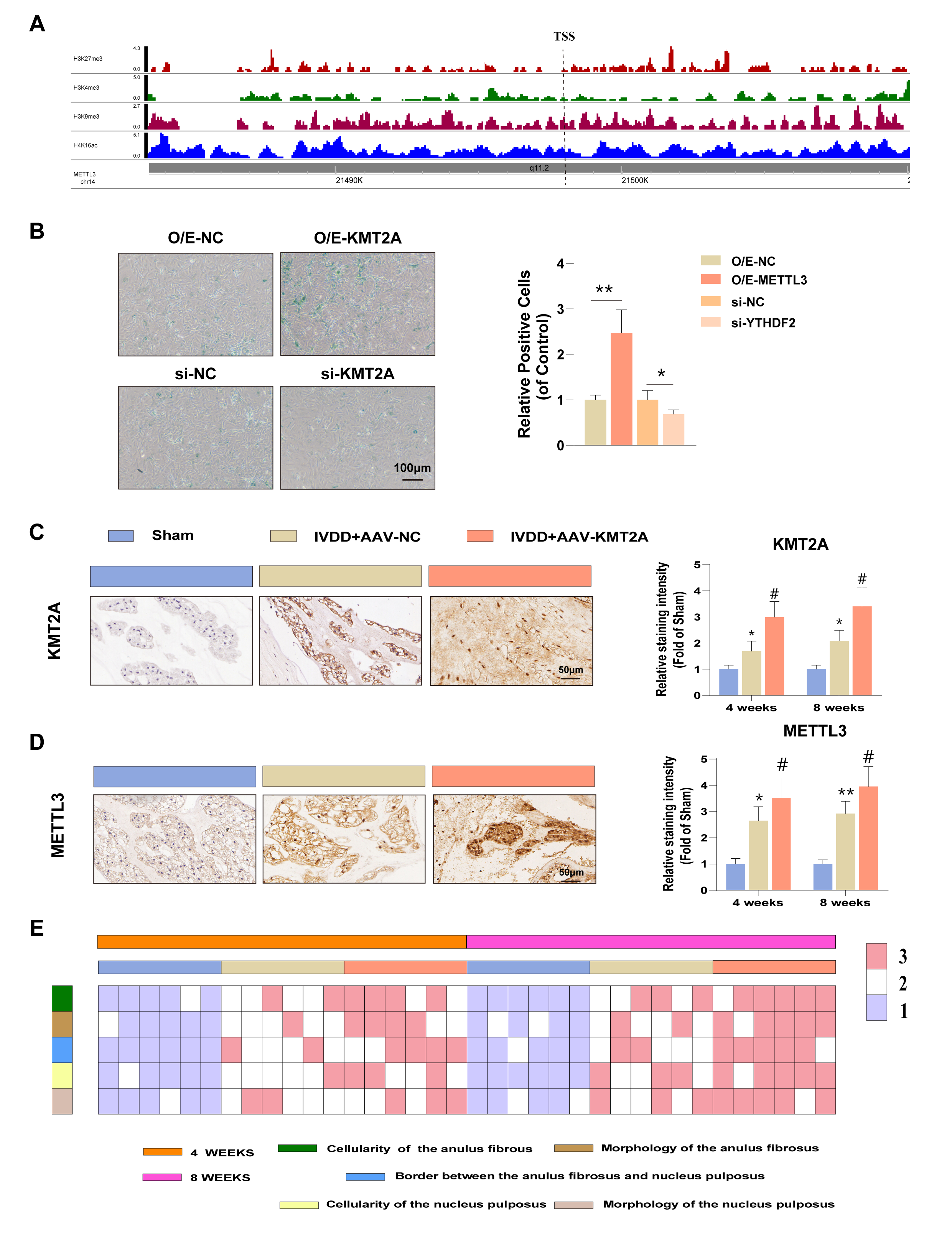


**Figure S11. (A)** Prediction of histone modifications in the METTL3 promoter using the WashU Epigenome Browser. **(B)** Number of positive cells after overexpression or silencing of KMT2A in normal NPCs by SA-β-gal assay (N = 3). *P < 0.05, **P < 0.01.**(C, D)** The respective IHC staining of KMT2A and METTL3 in NP tissues (N = 6). *P < 0.05, *P < 0.01, * VS Sham; #P < 0.05, # VS IVDD+AAV-NC. **(E)** Histological scores of mose NP tissues. Data are expressed as mean ± SD.


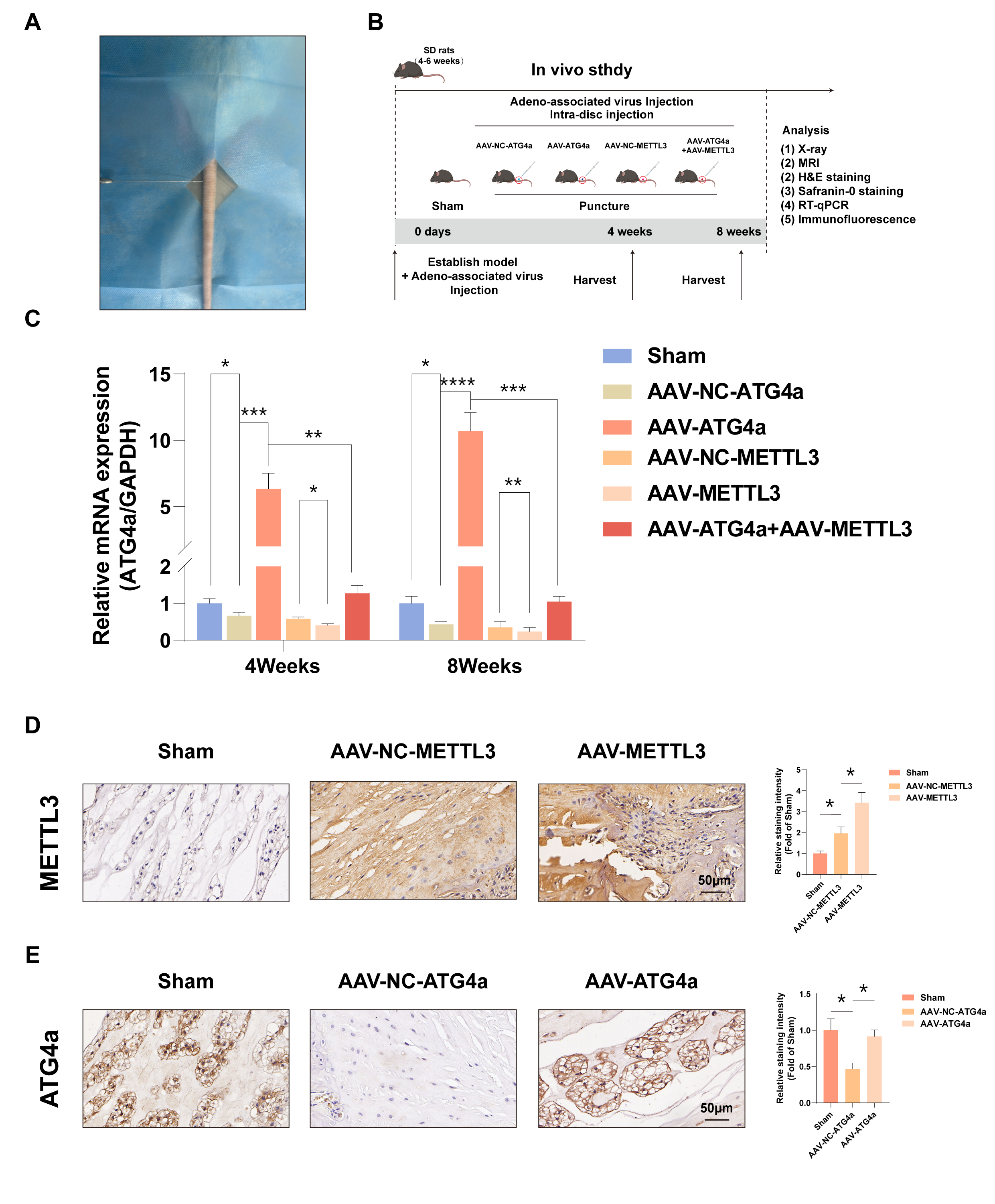


**Figure S12. (A)** The IVDD rat model was established by acupuncture. **(B)** Schematic illustration of IVDD model establishment and experiment design to evaluate the effects of ATG4a and METTL3 in vivo. **(C)** qPCR analysis of mRNA levels of ATG4a in NP tissues of rat tail spine at 4 and 8 weeks of injection (N = 3). *P < 0.05, **P < 0.01, ***P < 0.001, ****P < 0.0001.**(D, E)** The respective IHC staining of METTL3 and ATG4a in NP tissues (N = 6). *P < 0.05. Data are expressed as mean ± SD.


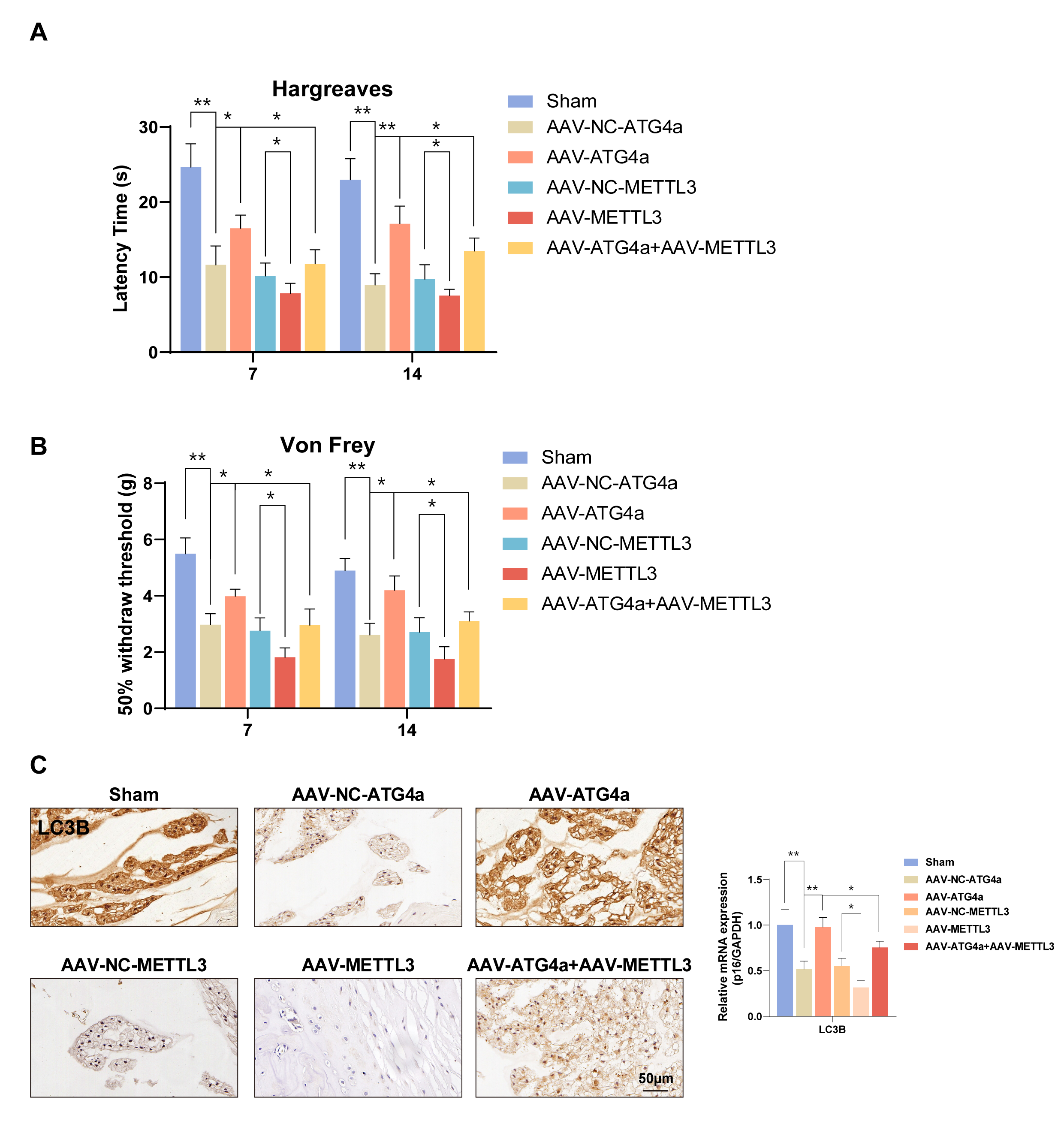


**Figure S13.** **(A)** Hargreaves test was performed to evaluate the thermal hyperalgesia of rats in various experimental groups (N = 6). *P < 0.05,**P < 0.01. **(B) V**on Frey test was performed to evaluate the mechanical allodynia of rats in various experimental groups (N = 6). *P < 0.05,**P < 0.01. **(C)** The respective IHC staining of LC3B in NP tissues (N = 6). *P < 0.05,**P < 0.01. Data are expressed as mean ± SD.


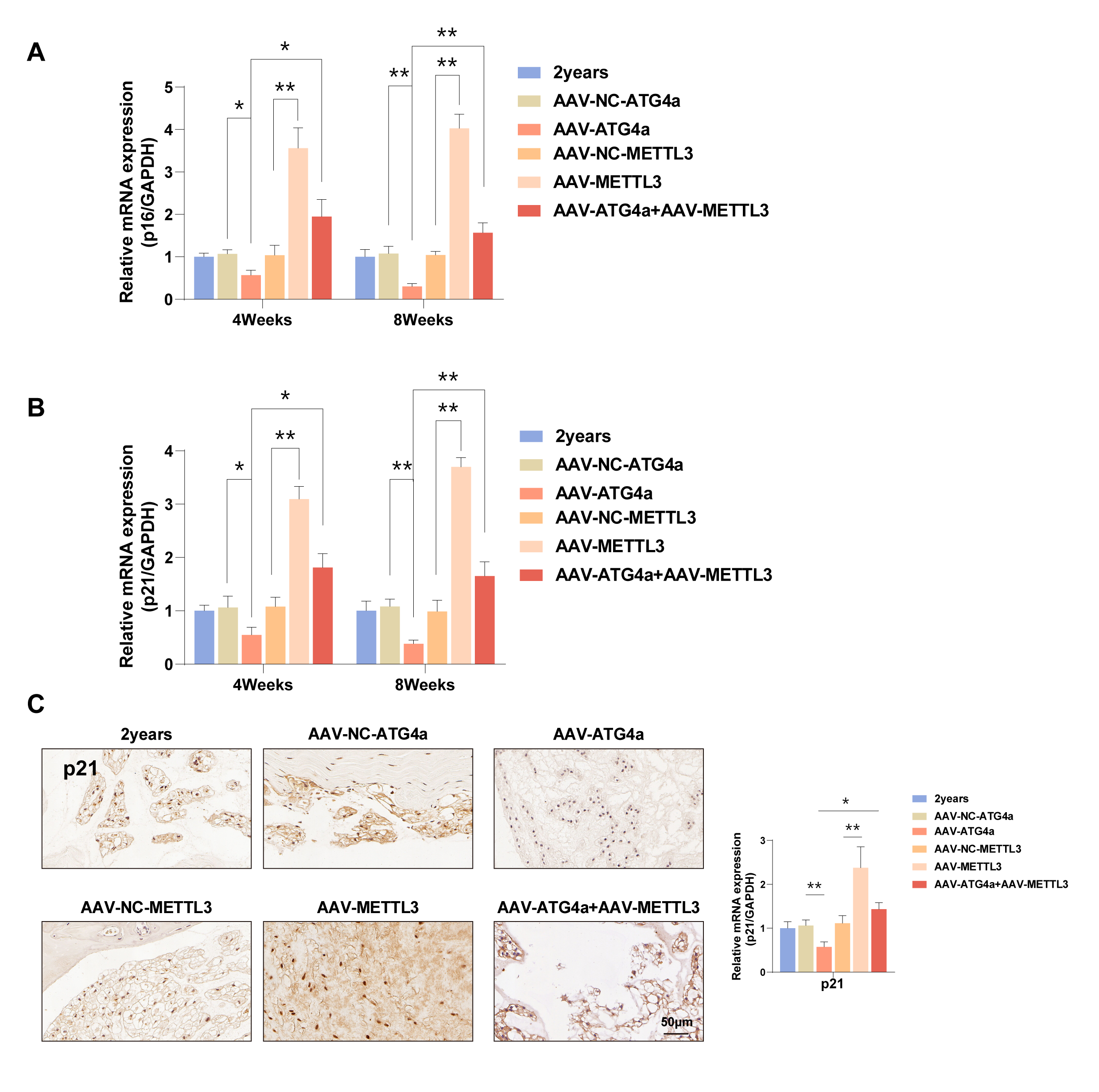


**Figure S14. (A)** qPCR analysis of mRNA levels of p16^INK4a^ in NP tissues of rat tail spine at 4 and 8 weeks of injection (N = 3). *P < 0.05, **P < 0.01. **(B)** qPCR analysis of mRNA levels of p21 in NP tissues of rat tail spine at 4 and 8 weeks of injection (N = 3).*P < 0.05, **P < 0.01. **(C)** The respective IHC staining of p21 in NP tissues (N = 6). *P < 0.05, **P < 0.01. Data are expressed as mean ± SD.
